# Calibration of head mounted displays for vision research with virtual reality

**DOI:** 10.1167/jov.23.6.7

**Published:** 2023-06-14

**Authors:** Nasif Zaman, Prithul Sarker, Alireza Tavakkoli

**Affiliations:** 1 Department of Computer Science and Engineering, University of Nevada, Reno, NV, USA

**Keywords:** virtual reality, color calibration, color vision

## Abstract

Immersion in virtual environments is an important analog for scientists. Situations that cannot be safely organized in the real world are being simulated virtually to observe, evaluate, and train aspects of human behavior for psychology, therapy, and assessment. However, creating an immersive environment using traditional graphics practices may create conflict with a researcher’s goal of evaluating user response to well-defined visual stimuli. Standard computer monitors may display color-accurate stimuli, but it is generally viewed from a seating position, where the participant can see real-world visual context. In this article, we propose a novel means to allow vision scientists to exert finer control over the participants visual stimuli and context. We propose and verify a device-agnostic approach to color calibration by analyzing display properties such as luminance, spectral distribution, and chromaticity. We evaluated five different head-mounted displays from different manufacturers and showed how our approach produces conforming visual outputs.

## Introduction

Recent advances in virtual reality (VR) head-mounted displays (HMD) have enabled wide adoption of the technology in diverse research areas. High-acuity screen resolution (Varjo XR-3, 2022), a wider field of view (Pimax 8k, 2022), higly affordable wireless VR (Oculus Rift, 2022), all-inclusive AR, VR, and eye-tracking capabilities (Vive Pro Eye, 2020) showcase how different commercial products have varied usefulness for research on fields such as vision science (Li et al., 2022), psychology (Ventura, Baños, Botella, & Mohamudally, 2018), therapeutics (Emmelkamp & Meyerbröker, 2021; Hilty et al., 2020), training (Kaplan et al., 2021; Lee, Park, & Park, 2019) and simulation (Stock, Erler, & Stork, 2018). Increasingly, researchers are adopting game engines such as Unreal Engine and unity to design and present immersive environments and stimuli to their subjects. Some of these applications require precise color specifications and display. However, no standard procedure exists that help researchers calibrate these HMDs and specify a color in their chosen color space for visualization.

Increasingly, VR technology is replacing traditional displays for more immersive experiments and assessments. For example, virtual simulation of ocular pathologies such as color vision deficiency (Cwierz, Díaz-Barrancas, Llinás, & Pardo, 2021), cataracts (Krösl et al., 2019; Krösl et al., 2020), and macular degeneration (Zaman, Tavakkoli, & Zuckerbrod, 2020) are helping researchers to quantify the effects of these diseases on quality of life. Additionally, VR-based visual assessments are being used to diagnose glaucoma (Skalicky & Kong, 2019), age-related macular degeneration (Zaman et al., 2020). Binocular compensation available in AR displays are being used to correct neuronal loss in experimental settings. Traditional optics may soon be replaced digital spectacles that manipulate the camera feed for recovery of visual function loss. However, such solutions in the fields of assessment, simulation, and rehabilitation would entail a module that can be easily calibrated for accurate color representation that is common for most medical usage of color displays.

Several lines of work exist that characterize the chromatic properties of different VR headsets and compare the perceptual performance with more traditional displays (Toscani et al., 2019), physical objective tests (Díaz-Barrancas, Cwierz, Pardo, Pérez, & Suero, 2020; Gil Rodríguez et al., 2022; Cwierz et al., 2021), and so on. In Diaz-Barrancas, Cwierz, Pardo, Perez, and Suero (2020), Díaz-Barrancas et al. (2020), and; Cwierz et al. (2021), the authors have implemented a way to reconstruct hyperspectral representation of physical scenes from multispectral images using CS-2000 tele-spectroradiometer. The reconstructed virtual scenes were Color-checker box, Ishihara test and Farnsworth-Munsell 100 Hue test. Although such a line of work is focused on creating an accurate virtual representation of captured scene components, in a separate line of work Kim, Cheng, Beams, and Badano (2021) and Toscani et al. (2019) have identified complex behavior in VR renders with Unity and Unreal Engine 4 (UE4), respectively, that invalidate standard color calibration practices. In Kim et al. (2021), the authors experimented with different render configurations to find the most perceptually correct representation for medical applications. In Toscani et al. (2019), the authors have disabled this behavior and formulated how to accurately control color and luminance in HTC Vive Pro Eye with Unreal Engine. However, their solution involved disabling system-wide tonemapping that is set by default in Unreal. Although this procedure causes clipped linear gamma behavior per channel and normal luminance additivity, it changes the behavior of many built-in shaders and materials, compromising the realistic effects default to Unreal Engine levels. Therefore, if researchers require fine-grained control over the behaviors of specific shaders without altering the appearance of the rest of the scene, a modular approach is necessary. More detailed differences between this work and ours will be discussed in the next section. In their subsequent work (Gil Rodríguez et al., 2022), the researchers showed the application of such a system to a real-world scenario, establishing the color constancy of a virtual scene calibrated using their approach. However, disabling the postprocessing routine means that the scene appears brighter and the light sources are clipped, contrary to the more realistic postprocess-enabled pathway. In Murray, Patel, and Wiedenmann (2022), the authors use look-up tables and color grading built into Unity to calibrate the luminance of VR headsets. Their procedure does not include color correction.

In this work, we present a framework for UE4 that calibrates and shows any viable color expressed in xyY, Luv, or Lab color spaces to the displays of a VR HMD in a modular manner. The main contribution of our proposed approach is the ability to present the scene such that parts of the view preserve default properties while specific stimuli behave according to specified chromaticity. Our packaged abstraction would allow researchers to create and render realistic stimuli without requiring extensive knowledge of the specific HMD device, spectrophotometer, or color representation in UE4. Furthermore, unlike Toscani et al. (2019), the proposed work would allow for a greater number of researchers to use VR technology for their workflow.

Our contributions in this work include measuring and comparing spectral distributions of major commercial HMDs including HTC Vive Pro Eye, Oculus Rift, Pimax, Fove and Varjo XR-3 using the i1 Pro 2 spectrophotometer (i1Pro, 2022). We build a novel UE4 camera asset that, when placed in any map, allows the scene to be processed along two different graphic pipelines. One pathway allows default Unreal rendering behavior to persist, so that objects in the scene appear realistic and provide a sense of immersion. The second pathway objects in the scene to display color-correct properties that are imperative for verifiable and reproducible vision research. This is achieved by modifying the postprocess material so that every object in the scene would go through the first pathway or if given a certain custom-depth stencil value would be processed along the second pathway. Furthermore, it works alongside i1Pro 2 to calibrate and preserve parameters of conversion for a specific HMD with regards to CIE xy, CIE Luv, and RGB. Finally, we validate whether the predicted and measured values of random color space coordinates match up closely.

## Materials and methods

Standard color calibration practices make some assumptions about the properties of the display. Similar to Toscani et al. (2019), we characterize the properties of the HMDs to apply the appropriate calibration protocol. Suppose, we define default postprocess tonemapping as a function of input emissive FLinear Color RGB values τ(*RGB*). FLinear Color is an Unreal Engine object that defines color within a range of [0,1]. This is distinct from the reflectance values considered in Toscani et al. (2019), where the scene objects were either illuminants or Default Lit shading materials and, therefore, depended on illumination and viewing conditions.Then, disabling tonemapping has the following effect on input emissive values:

(1) $$\begin{array}{c} {\bar{Lxy}}_{view}=\tilde{\tau }\left(RGB\right),\; \end{array}$$

where $\tilde{\tau }$ is a composite postprocess that includes all other postprocess operations except for tonemapping τ. In our setup, we use self-emitting virtual surfaces and we do not disable system wide tonemapping, and instead, use a mapping function α(*RGB*) that converts stimuli RGB values so that, for the output chromaticity,

(2) $$\begin{array}{c} {\bar{Lxy}}_{stimuli}=\tilde{\tau }\left(\tau \left(\alpha \left(RGB\right)\right)\right)\; \end{array}$$

has the same effect as $\tilde{\tau }$, while the rest of the scene behaves as default:

(3) $$\begin{array}{c} Lxy_{scene}=\tilde{\tau }\left(\tau \left(RGB\right)\right)\; \end{array}$$

Scene objects are any part of the three-dimensional environment that is rendered using the Default Lit shading model and, therefore, is influenced by external lighting, shadow, tonemapping, and other postprocesses. Stimuli are objects that have no inherent chromaticity and obtain their color value from prescribed *Lxy* values that are calibrated to display exact chromaticity output to the HMD screen.

### Experimental setup

Our experimental setups include a handheld spectrophotometer for recording HMD display outputs. The spectrophotometer is controlled using MATLAB script and the HMDs are controlled using Unreal Engine. We placed the spectrophotometer against the right display of the HMDs. The device was held in place so that it pointed to the center of the display. As we did not have access to wide field colorimeters such as the I29 used by Gil Rodríguez et al. (2022), this may have resulted in off-axis measurements. We make comparisons between the characteristics of the separate HMDs and between the left and right ocular displays to help establish reliable color and luminance calibration procedures.

### Calibration process

For calibration, two sets of data were collected for each headset. The first set involved measuring luminance and chromaticities of ten linearly spaced points in the R = RGB(*x*, 0, 0), G = RGB(0, *x*, 0), and B = RGB(0, 0, *x*) channels as well as a combined channel (*x*, *x*, *x*) where *x*ϵ{0, 1}. The latter set involved measuring spectral distributions (*S*_*p*_), chromaticities (*x*, *y*), and luminance (*Y*) from red, green and blue primaries alongside the white point.

Using the first set of measurements we modeled the gamma correction, relation between input RGB to the emissive property of the shaders in Unreal Engine and the output luminance *Y*. These relations were established for three different scenarios:

• Conventional postprocessing.
• Postprocess tonemapping disabled.
• Selective correction.

With the second set of measurements we verified the established relation between the input RGB and the chromaticity of the output (*x*, *y*).

### Considerations before rendering in unreal engine

**Figure 1.**
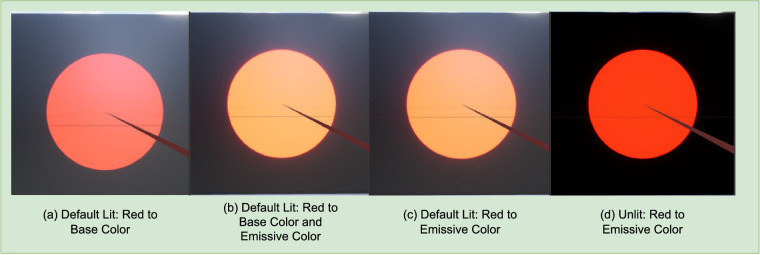
Comparison between different shading models and attributes.

Rendering in Unreal Engine entails several considerations that help to avoid unintended color effects. A scene in Unreal Engine consists of two types of objects: illumination sources and reflective objects. Unlit shaders behave the same way under any lighting condition and can be considered as a self-emissive surface material. Emissive attribute is the only component that produces chromaticity and luminosity in unlit shaders. Default-lit materials, in contrast, are affected by lighting conditions. Figure 1 demonstrates these differences. After a main shading pass, which applies lighting and specular properties to objects in default-lit mode and only emissive property in unlit mode, the scene is passed through a series of post processing steps that introduce effects like tonemapping, motion blur, flare, and bloom. Selecting unlit shader model does not have any impact on these postprocess steps because these attributes are not influenced by external lighting conditions, but by their own properties of self-emission. Therefore, to correct any tonemapping and color grading, a method can either be setup by placing an unbounded postprocess volume in the scene that impacts every scene object within render view (Toscani et al., 2019) or by changing the postprocess material of the scene camera. We chose to create a blueprint ready object (PostCamera) that inherits from the Camera object in Unreal Engine and is modified to disable all postprocessing steps solely through camera settings when placed within a scene and used as target view. Additionally, for rendering and measuring stimuli properties, we chose to disable all illumination sources except for the stimuli. The stimuli material has lambertian surface parameters enabled with emissive (RGB) values set to be visible to the scene camera. This material is applied to a square plane directly in front of the scene camera. Moreover, our approach allows users to visually represent two kinds of materials at once: i) material displaying conventional photorealistic properties and ii) material displaying color-correct properties. By assigning selected specific values to the custom depth pass for each object in the scene, photorealism-specific graphics routines are not applied to them. This means illumination, shadow, exposure, and so on, have no effect on the colorcorrect material rendered to the corresponding final HMD pixel output due to its separate graphics pipeline.

**Figure 2.**
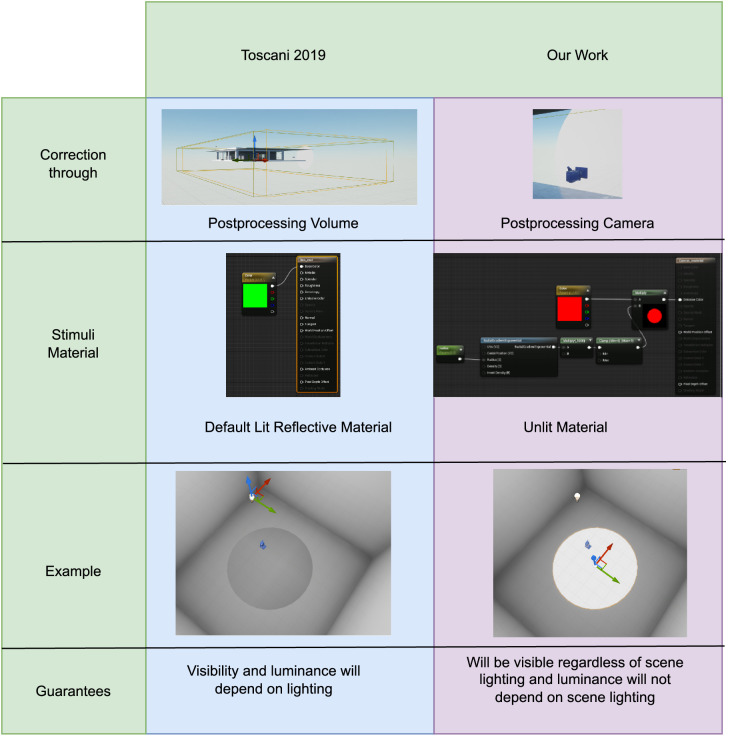
Comparison between (Toscani et al., 2019) and our approach.

In some classical approaches, the light source of the scene is manipulated to change the reflectance value of materials for the desired HMD output. Generally, this approach would be ineffectual in Unreal Engine rendering pipeline as the postprocess routines would still alter the chromaticity and luminance intensity of the scene objects. To make this classical approach work, Toscani et al. (2019) disabled the complete postprocess routines. However, this approach still has some limitations. First, after calibration with a certain illumination, the scene illumination needs to remain same for all scenes that use the calibrated reflectance values. Instead, in our approach, we leverage the postprocess pathway in such a manner that objects with custom depth pass assigned to them will behave not as default lit shader materials, but as self-illuminants. By leveraging emissive property and the postprocess pathway, we ensure that the rendering results are final and independent of scene illumination. Thereby, calibration process does not need to be repeated if the scene illumination changes. Figure 2 summarizes the important differences between Toscani et al. (2019) and our work.

### Equipment

For measuring display outputs we use the i1Pro 2 (i1Pro, 2022) spectrophotometer. We measure the HMDs mentioned in Table 1. The data in Table 1 are taken through independent measurements (Murphy, Maraj, & Hurter, 2018; Hmd geometry database, 2021) or manufacturer reports (Hmd comparison, 2022). Most VR devices have masked diagonal regions, decreasing the diagonal visible area from those reported by manufacturers. Therefore, the pixel densities were calculated by dividing horizontal resolution with horizontal field of view.

**Table 1.** Specifications of VR headsets.

	**Rendered field of view (horizontal in degrees)**	**Display type**	**Pixel density (pixel per degrees)**	**See-through camera**	**Native eye tracking**
**HTC Vive Pro Eye**	107	AMOLED	13.45	Yes	Yes
**Oculus Rift**	88	OLED	12.27	No	No
**Fove 0**	90	OLED	14.2	No	Yes
**Pimax 5k Plus**	160	LCD	16	No	No
**Varjo VR-3**	115	uOLED (center) LCD (peripheral)	70 (central) 30 (peripheral)	Yes	Yes

The HTC Vive Pro Eye is widely used for its all-round functionalities which include a moderate resolution, field of view, eye tracking and video see-through camera (480 p). Oculus Rift has lower resolution (1,440 vs. 1,080 horizontal per eye) and field of view, but is a good choice for commercial therapeutics because of the affordability of the Oculus headsets. Pimax 5k Plus has a considerably wider field of view and resolution (2560 horizontal per eye). Fove 0 is a compact alternative to the HTC Vive Pro Eye. It has a limited field of view and resolution (1,280 horizontal per eye). However, its eye tracking api allows vision researchers to monitor external ocular properties in real time. The Varjo VR-3 is a considerable improvement over all the other HMDs discussed so far. It has the highest resolution displays (central 27 degrees 1,920 and peripheral 88 degrees 2,880 horizontal per eye), and cameras (1,080 p). It has an eye tracking api that gives functional access similar to Fove. However, its cost may make it unsuitable for some research and commercial assessment and therapeutics. These devices vary in display types (AMOLED: HTC Vive Pro, OLED: Fove 0, Oculus Rift, LCD: Pimax 5k and mixed: Varjo VR-3). The mixture of two different display types in Varjo VR-3 of uOLED (central 27°) and LCD (peripheral) requires additional consideration before application in color vision research. The applications were rendered in a computer with a 4.2-GHz Intel Core i7 processor and a Nvidia GeForce RTX 2080 graphics card.

## Rendering with conventional postprocessing

This is the default behavior in UE4.27. In these set of experiments, all camera and postprocessing settings were left to default behavior.

### Relationship between input intensity and luminance

In Unreal Engine, instances of light sources such as point light, and directional light have intensity values (cd and lux respectively) associated with them. However, physically based lighting may have unintended effects on stimuli color perception. Therefore, we chose to use the emissive values (RGB) of the stimuli material to reproduce the intended luminance (Y). This procedure allows stimuli chromaticityperception to be independent of scene illumination and context although stimuli perception will still be affected.

**Figure 3.**
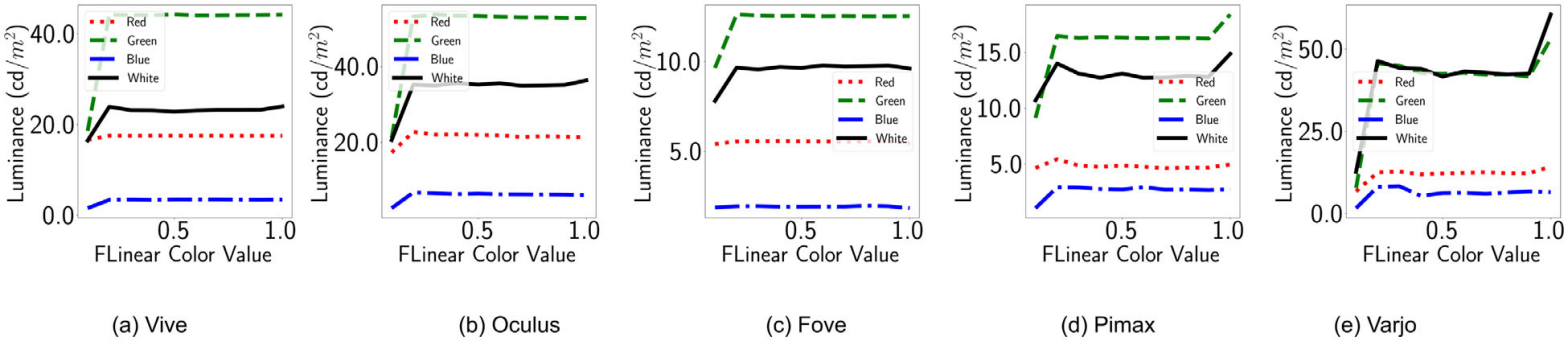
Default configuration. The X axis shows emissive values *x* in scene material: *RGB*(*x*, 0, 0) for red, *RGB*(0, *x*, 0) for green, *RGB*(0, 0, *x*) for blue, and *RGB*(*x*, *x*, *x*) for white. The Y axis shows luminance in *cd*/*m*^2^.

Figure 3 shows the luminance (cd/m^2^) corresponding to the input R = RGB(*x*, 0, 0), G = RGB(0, *x*, 0) and B = RGB(0, 0, *x*) emissive for the HMDs. The figures demonstrate how for different HMDs the luminous intensities are different for the same emissive values. LED LCD displays can be brighter than variants of OLED displays. However, this is not evident in the results shown in Figure 3. As we shall see in the other variants of this experiments, Unreal Engine’s postprocess routines play a role in the diminished brightness of LED LCD displays. Furthermore, the default camera exposure lead to the intensity of emissive materials in the scene to saturate quickly. Here, our observations differ dramatically from the ones reported by Toscani et al. (2019) because they simply used default shaded materials and reflectance values, whereas we used emissive materials. Default emissive materials behave like light sources and reach a predefined intensity to emulate light exposure. For this reason, in subsequent methods, we disabled the autoexposure property. Further, in Figure 3, we see that green has higher luminance than white, which is also a result of this autoexposure property of self-emitting surface materials.

All the displays now exhibit properties of a clipped linear function. The relationship is modeled as follows:

(4) $$\begin{array}{c} L=\left\{\begin{array}{cc} x\cdot m_{X\varepsilon [R,G,B]}, & x\cdot m_{X}<t\\ t, & otherwise \end{array}\right.\; \end{array}$$

where L is the luminance, *m*_*X*_ is the slope corresponding with a specific channel, and *t* is the threshold beyond which the luminance does not vary with change in *x*. Table 2 shows the corresponding values of *m*_*X*_, *t* for each HMD.

**Table 2.** Default configuration: Slope and threshold for modeled relationship between luminance and emissive value.

	HTC Vive		Oculus		Fove		Pimax		Varjo	
	*m*_*X*_	*t*	*m*_*X*_	*t*	*m*_*X*_	*t*	*m*_*X*_	*t*	*m*_*X*_	*t*
Red channel	8.9	17.5	54.6	22.7	1.5	5.5	7.8	5.4	57.6	12.5
Green channel	255.6	44.15	319.8	53.1	29.8	12.6	73.4	16.5	379.7	45.6
Blue channel	18.3	3.36	41.3	6.75	0.6	1.93	18.7	2.9	65.2	8.1

Table 2 shows that highest exposure effect is displayed by Varjo for green colors. Varjo is also the brightest in this default configuration, while Fove is the dimmest. Therefore, vision research that involves scotopic stimuli, with realistic rendering still enabled, can make use of Fove for their research. In contrast, for simulation of real-world performance, Varjo is ideal, because it exhibits the highest dynamic range (signified by the steep slope). However, HTC Vive can be a good alternative if affordability is a concern.

All displays show a spike and saturation at the start, which is caused by the saturation due to camera exposure. Pimax and Varjo HMDs show a secondary spike. In Varjo, white is the brightest, whereas in all other displays, green is the brightest. The OLED displays (Oculus and Fove) show equal spread between the luminance of these colors. Fove and Pimax have considerably dimmer brightness compared to the other HMDs. As default gamma correction is enabled, displays show piece-wise linear behavior.

### Luminance additivity

**Figure 4.**
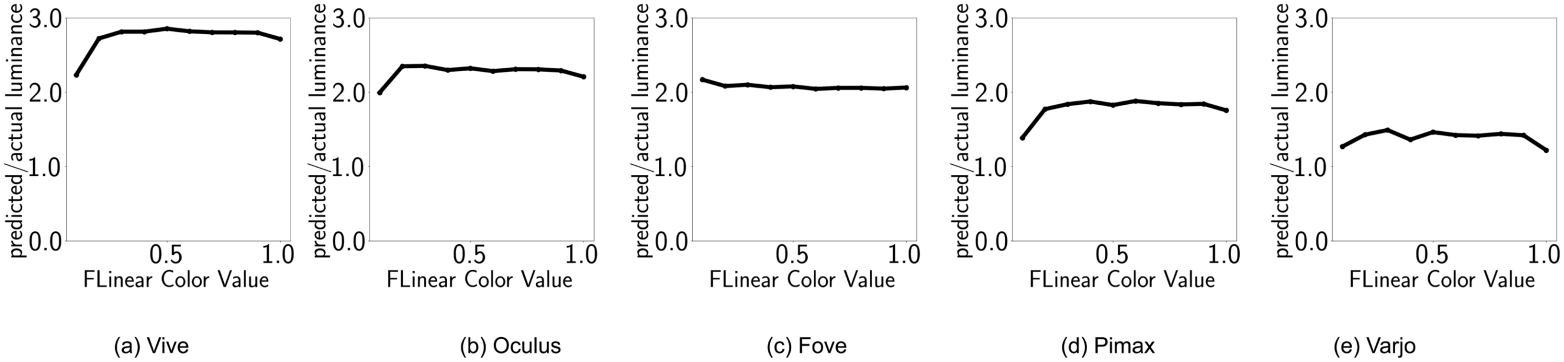
Default configuration. The X axis shows emissive values x and the Y axis shows the ratio (*L*_*R*_ + *L*_*G*_ + *L*_*B*_)/*L*_*W*_.

For standard displays, summation of *R*, *G*, and *B* channel intensities give grayscale intensities:

(5) $$\begin{array}{ccc} & & L(RGB(x,x,x))=L(RGB(x,0,0))\\ & & \;+L(RGB(0,x,0))+L(RGB(0,0,x))\; \end{array}$$

demonstrating additive property. However, when postprocess tonemapping is enabled, as is default for UE4, such behavior is not visible (Figure 4). Instead, subadditive property is observed for all HMDs. This shows considerably more subadditivity (approximately 200%) compared with the one observed by Toscani et al. (2019) because of our choice of self-emitting surfaces. Here, we can see that Fove is the only device that shows near constancy across *x* values. All other devices show an upfront increase in ratio (caused by exposure) and a dip at the tail (for *x* = 1, *L*_*W*_ transitions from grey to white). For different devices, the ratio of predicted (sum of individual channels) and measured luminance are shown in Table 3. It further shows that Varjo is closest to displaying luminance additivity while HTC Vive is the farthest. It means, for HTC Vive, white is significantly less bright than the summation of the primaries.

**Table 3.** Default configuration: Average luminance ratios for ten shades within *x*ϵ[0.1, 1.0].

	HTC Vive	Oculus	Fove	Pimax	Varjo
(*L*_*R*_ + *L*_*G*_ + *L*_*B*_)/*L*_*W*_	2.74	2.27	2.07	1.78	1.39

### Channel constancy

Channel constancy is maintained when scaling emissive values (RGB values) linearly also scales the spectral outputs by the same factor. Channel constancy means that scaling the channels independently would not change the chromaticity and only impact luminance. To determine whether color constancy is preserved for all HMDs, we carry out the following experiment. We measure the spectral output (ϕ(*RGB*)) for *x* = 0.1 in R = RGB(*x*, 0, 0), G = RGB(0, *x*, 0), and B = RGB(0, 0, *x*) channels separately. Next, we obtain spectral outputs (Radiance $\frac{W}{sr\cdot m^{2}}$) for *x*ϵ[0.1, 1.0] for all the channels. If the spectral outputs are a linear scaling of *x*, such that:

(6) $$\begin{array}{c} \phi_{x=[0.1,1.0]}^{X}=c\cdot \phi_{0.1}^{X}\text{;}\;\text{where}\;X\varepsilon \left[R,G,B\right],\; \end{array}$$

then it can be said that the HMDs maintain channel constancy. For the only HMD with more than one type of display (Varjo with uOLED and LCD), the channels show multiple peaks for the primaries (Figure 6). The local peaks indicate multiple wavelengths are dominantly present in the composition of the primaries. For the rest of the HMDs the channels show a single peak. Moreover, the channels have the same scaling factor of 1 for all the devices except for Varjo. This means only Varjo violates channel constancy. Using the scaling factor (*c*) of the peak of each shade and 0.1 shade we calculate the mean error in Table 4. The error, *e* is calculated by taking the mean square error of all $c*\phi_{0.1}^{X}$ and $\phi_{x}^{X}$ This negligible error demonstrates good channel constancy for all other devices. For Varjo the constancy error is still small, as we used the peak ratio

**Table 4.** Channel constancy scaling error $mse(c*\phi_{0.1}^{X},\phi_{x}^{X}$).

	HTC Vive	Oculus	Fove	Pimax	Varjo
*c*_*red*_	0.001	0.002	0.002	0.002	0.005
*c*_*green*_	0.02	0.007	0.003	0.002	0.017
*c*_*blue*_	0.04	0.006	0.002	0.005	0.023

**Figure 5.**
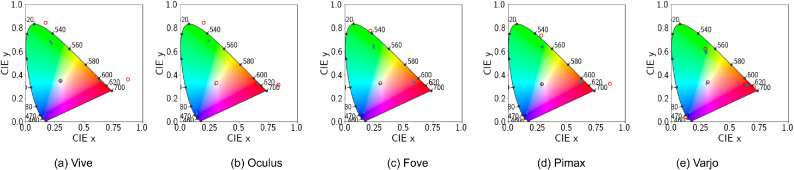
Default configuration. CIE 1931 Chromaticity diagram with ‘x’ denoting actual device output for different shades (0.1 through 0.9) of the primaries. ‘o’ denotes the theoretical position of the actual primaries using the calibration matrix.

**Figure 6.**
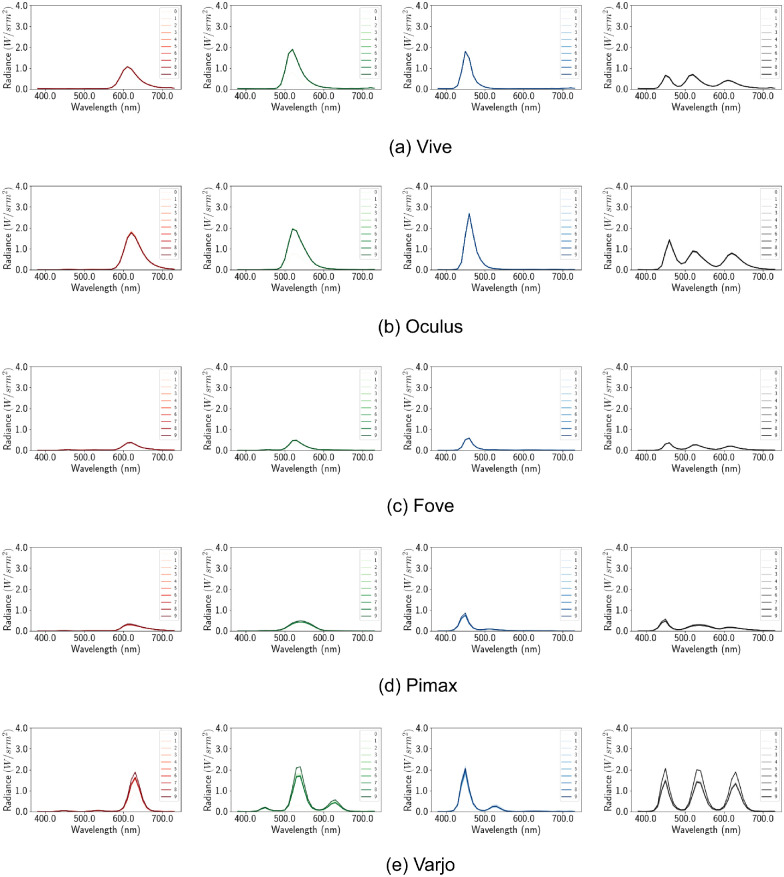
Default configuration. Per channel spectral distribution graph. The X axis denotes the wavelength in nanometers and the Y axis denotes the spectral output at that wavelength. The spectral distribution of different shades of red, green, blue, and white (from left to right) are shown for each device (top to bottom).

However, this may simply be caused by auto exposure transforming intermediate brightness levels to the primary brightness level. Further violations of channel constancy is demonstrated in Figure 5. Only the Varjo HMD primaries fall inside the chromaticity coordinates while displaying a slight drift in the shades. For all the rest of the HMDs, the calibration process creates a calibration matrix that when applied to original primary RGB values in R = RGB(1, 0, 0), G = RGB(0, 1, 0), and B = RGB(0, 0, 1) output chromaticity values that fall outside the gamut. This means the calibration process based on autoexposure peak values is faulty We will further investigate the calibration process in the following subsection.

### Calibration test

For standard color calibration to be applicable, luminance additivity and channel constancy must be preserved (Brainard, 1989). However, as we have seen in the preceding sections, these properties are not demonstrated by any of the VR devices while Unreal Engine’s default postprocess tonemapping is enabled. This is further demonstrated when we try to use standard calibration practices to this system. We record the primaries and using the least square method find an *M* that minimizes the euclidean distance *XYZ* − *M* · *RGB*^*T*^. Original *XYZ* are obtained from the spectroradiometer measurements of the primaries by converting *xyY* to *XYZ*:

(7) $$\begin{array}{c} X=x*Y/y\; \end{array}$$

(8) $$\begin{array}{c} Y=Y/100\; \end{array}$$

(9) $$\begin{array}{c} Z=(1-x-y)*Y.\; \end{array}$$

**Figure 7.**
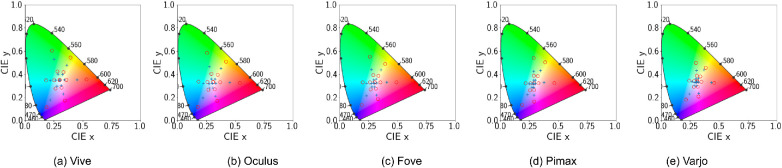
Default configuration. CIE 1931 Chromaticity diagram with ‘x’ denoting actual device output for different points on the cubes discussed earlier.‘o’ denotes the theoretical position of the actual RGB values using the calibration matrix.

To verify if the calibration was successful, we display eight different emissive values. These emissive values correspond to the eight corners of two cubes such that one corner of the bigger cube is located at RGB(0.2, 0.2, 0.2) and the other corner at RGB(0.8,0.8,0.8) and one corner of the smaller cube is located at RGB(0.4,0.4,0.4) and the other corner at RGB(0.6,0.6,0.6). Essentially, these combination of colors are selected simply because of their spread in both RGB and chromaticity space (Figure 7) which facilitates verification of the calibration process. Next, we measure the *Lxy* values with the spectroradiometer. Let us represent the corresponding CIE xy chromaticity values as *Lxy*_*measured*_. Using the conversion matrix from the primaries, let us now obtain the calibrated position of the cube corners:

(10) $$\begin{array}{c} Lxy_{predicted}=Lxy\left(M\cdot RGB_{cube}^{T}\right)\; \end{array}$$

Figure 7 shows how the plotted *Lxy*_*measured*_ and *Lxy*_*predicted*_ deviate, confirming that standard calibration is not effective. The theoretical coordinates of the cube points vary significantly between devices owing to the variation in the chromaticity of the primaries measured with the spectroradiometer. This is because we use those values to construct the conversion matrix *M*. Because the Varjo device had the primaries most closely situated with the theoretical position, the mapped cube points also are quite near to the actual device measurement.

In the following sections we will demonstrate two methods to calibrate and display nominal values by 1) disabling tonemapping and 2) computationally correcting for tonemapping.

## Rendering with postprocess tonemapping disabled

**Figure 8.**
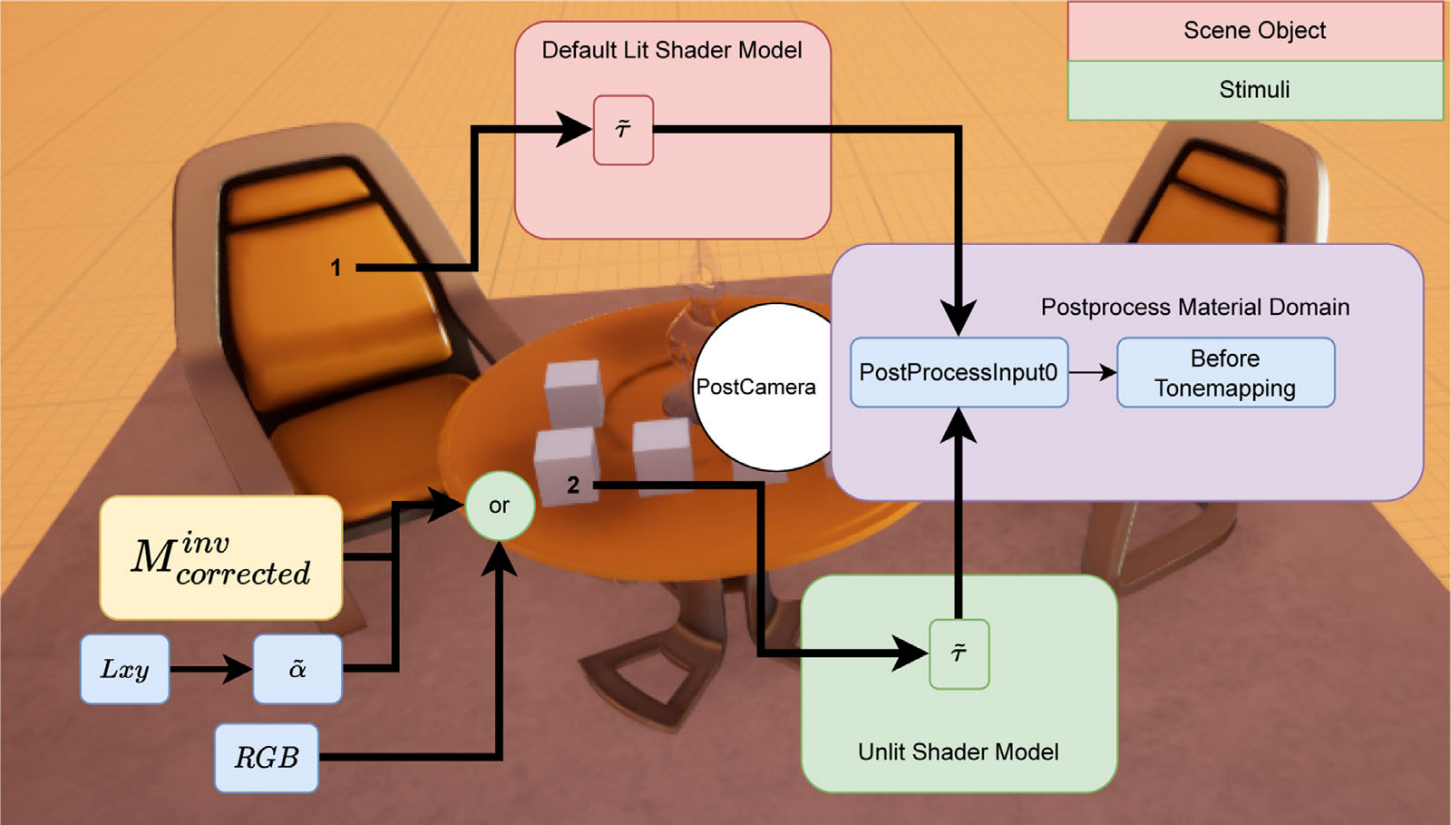
Demonstration of rendering pipelines for scene object and stimuli object.

As we saw in the earlier section, the standard camera in Unreal Engine had auto exposure enabled by default which was responsible for the discrepancy with luminance additivity, and channel constancy properties needed for accurate color calibration. These effects were not present in Toscani et al. (2019) as their objects were reflective instead of self-illuminant. We therefore supplanted the standard camera with our PostCamera object. Our PostCamera object inherits from the camera actor object in Unreal Engine. It is setup to partially disable tonemapping at begin play. Disabling tonemapping entails overriding the autoexposure, bloom, motion blur, grain jitter, scene fringe, and graining. Additionally, we applied a postprocess material that altered the blendable location to Before Tonemapping. In Unreal Engine, blendables are sets of parameters such as base color, opacity, and so on that are passed on to graphics pipeline for rendering. Different stages of the rendering pipeline read and write to different blendables. When set to Before Tonemapping, PostProcessInput0 in the postprocess material editor provides access to scene color with all lighting HDR. Therefore, we use it to only counter the effects of tonemapping using a single pipeline for both stimuli and scene objects. Figure 8 shows the rendering pipeline and how the input emissive or chromaticity values are interpreted to render accurate pixels to the HMD. Now with these altered settings, we repeated the previous experiments.

### Relationship between input intensity and luminance

**Figure 9.**
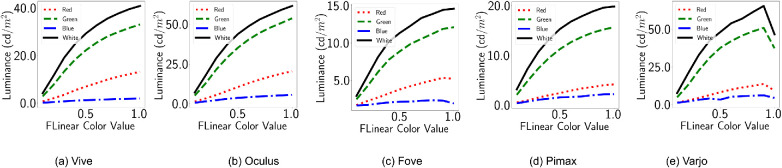
Disabled tonemapping. The X axis shows emissive values x in *RGB*(*x*, 0, 0) for red, *RGB*(0, *x*, 0) for green, *RGB*(0, 0, *x*) for blue, and *RGB*(*x*, *x*, *x*) for white. The Y axis shows luminance in *cd*/*m*^2^.

Figure 9 shows the luminance (cd/m^2^) corresponding to the input R, G and B channels. The figures demonstrate how different HMDs react to disabling postprocess tonemapping. As the auto exposure is now disabled, the piece-wise-linear relation is not present. Instead, we can see that disabling the tonemapping has disabled gamma correction as well. This is unlike the relationship found by Toscani et al. (2019) where they found piecewise-linear relationship restored by the same setting. It is possible that their settings included additional routines to enable gamma correction. Otherwise, the luminance groups are conserved so that Fove and Pimax are still dimmer than the rest. Instead of the tail spike that was visible for Varjo with default settings, now the opposite can be seen. The primaries seem to be dimmer compared with a portion of the shades of the primaries.

This also illustrates a significant limitation of the normal settings in Unreal Engine. If a stimulus required precise luminance control, that cannot be attained as auto exposure maps most intensities over a threshold to saturation. Using this disabled tonemapping state, intermediate luminance levels can be reached.

### Luminance additivity

**Figure 10.**
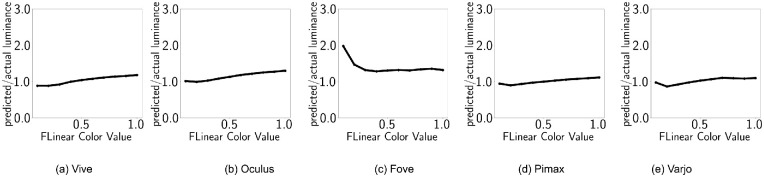
Disabled tonemapping. The X axis shows emissive values x, while the Y axis shows the ratio (*L*_*R*_ + *L*_*G*_ + *L*_*B*_)/*L*_*W*_.

Figure 10 shows the luminance additivity property discussed earlier. The dashed lines represent predicted luminance (summation of channel luminance) and the circles represent actual grayscale luminance. As the predicted and measured luminance values align, we can say that the luminance additivity property is preserved. For different devices, the ratio of predicted (sum of individual channels) and measured luminance are shown in Table 5.

**Table 5.** Disabled tonemapping: Average luminance ratios for ten shades within *x*ϵ[0.1, 1.0].

	HTC Vive	Oculus	Fove	Pimax	Varjo
(*L*_*R*_ + *L*_*G*_ + *L*_*B*_)/*L*_*W*_	1.04	1.14	1.39	1.01	1.02

The ratios are now much closer to 1.0. However, the OLED display (Oculus and Fove) ratios are slightly higher. Additionally, Fove HMD shows the same drop it exhibited in the normal settings.

### Channel constancy

As with the earlier channel constancy experiment, here we determine whether spectral distributions at higher emissive values are some constant multiples of the ϕ(*x* = 0.1) distributions.

Compared with single spectral distributions for single type displays, the new distributions have separate distributions for each shade of the primary colors (Figure 12). This is a direct result of turning off auto exposure, as now different shades have different brightness and therefore different peaks in their spectral distribution. Furthermore, the scaling factors *c* seem to denote multiplicative spectral profile and channel constancy. Using the same methods of the earlier section we calculate the mean squared error with the scaling factors shown in Table 6.

**Table 6.** Disabled tonemapping: Channel constancy scaling error.

	HTC Vive	Oculus	Fove	Pimax	Varjo
*c*_*red*_	0.017	0.028	0.069	0.01	0.078
*c*_*green*_	0.019	0.018	0.058	0.017	0.047
*c*_*blue*_	0.012	0.013	0.056	0.007	0.052

**Figure 11.**
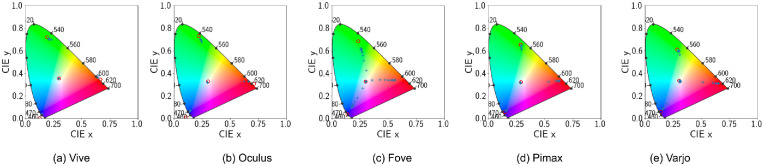
Disabled tonemapping. CIE 1931 Chromaticity diagram with ‘x’ denoting actual device output for different shades (0.1 through 0.9) of the primaries. ‘o’ denotes the theoretical position of the actual primaries using the calibration matrix.

Because auto exposure is now disabled, we can more easily visualize the difference in luminance for each shade. Preservation of channel constancy is also demonstrated in Figure 11. Fove shows significant drift in measured chromaticity for shades of the red, green, and blue colors. Other HMDs show negligible shift in chromaticity. This slight drift is caused solely by uncorrected gamma. Color gamut for Pimax and Varjo are also within the boundary of the coordinate system. Demonstrating that disabling tonemapping is a much better solution for visual stimuli presentation in VR-based vision science products.

### Color calibration test

We record the new primaries and using the least square method find an *M* that minimizes the euclidean distance *XYZ* − *M* · *RGB*^*T*^. New *XYZ* are obtained from the spectroradiometer measurements of the primaries by converting *xyY* to *XYZ*.

Figure 13 shows the positions of the cube corners in CIE XY space for predicted and measured xyY values with the spectroradiometer. Let us represent the corresponding CIE xy chromaticity values as *Lxy*_*measured*_. Using the conversion matrix from the primaries, let us now obtain the calibrated position of the cube corners:

(11) $$\begin{array}{c} Lxy_{predicted}=Lxy\left(M\cdot RGB_{cube}^{T}\right).\; \end{array}$$

**Figure 12.**
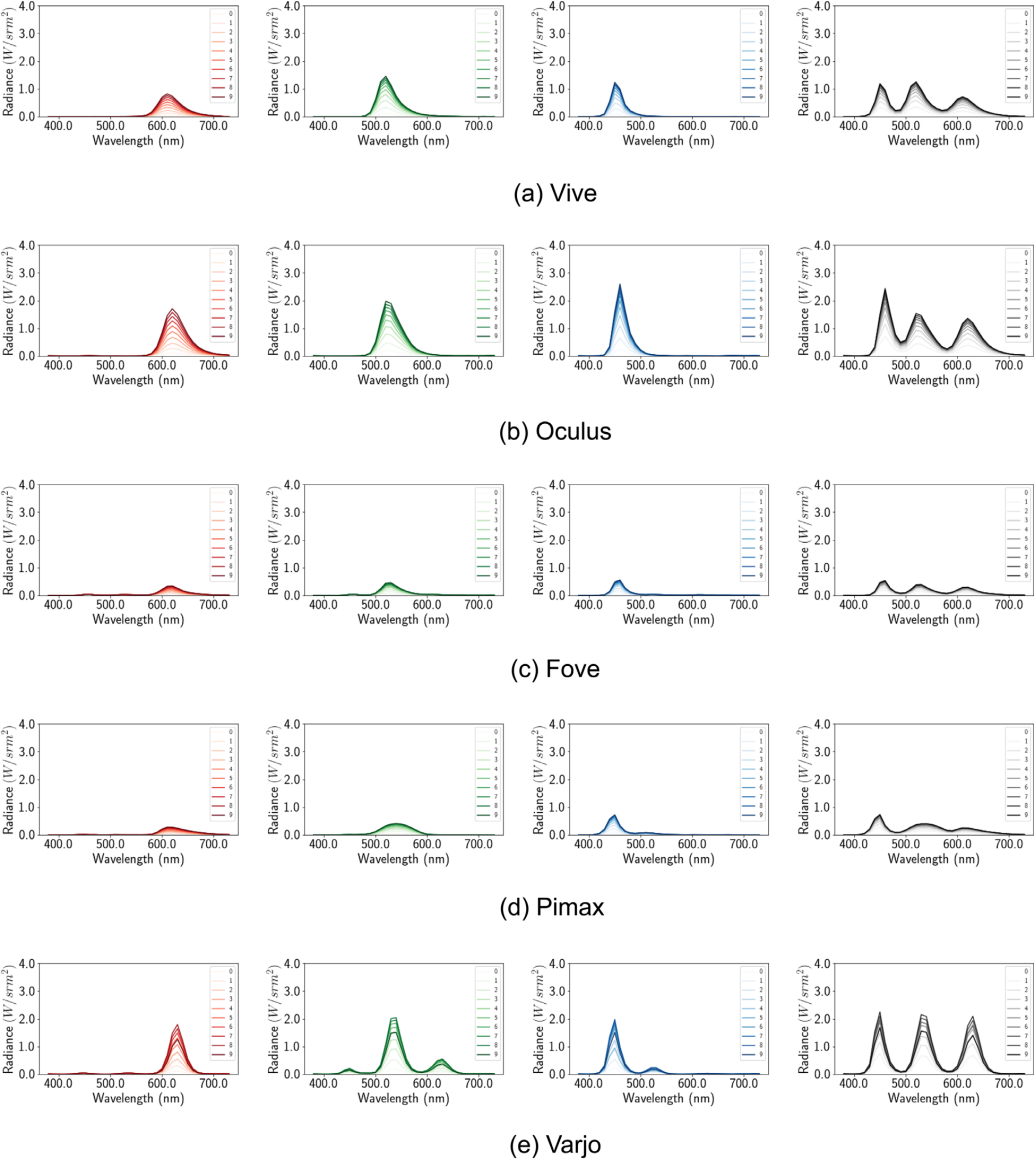
Disabled tonemapping. Per channel spectral distribution graph. The X axis denotes the wavelength in nanometers and the Y axis denotes the spectral output at that wavelength. The spectral distribution of different shades of red, green, blue, and white (from left to right) are shown for each device (top to bottom).

**Figure 13.**
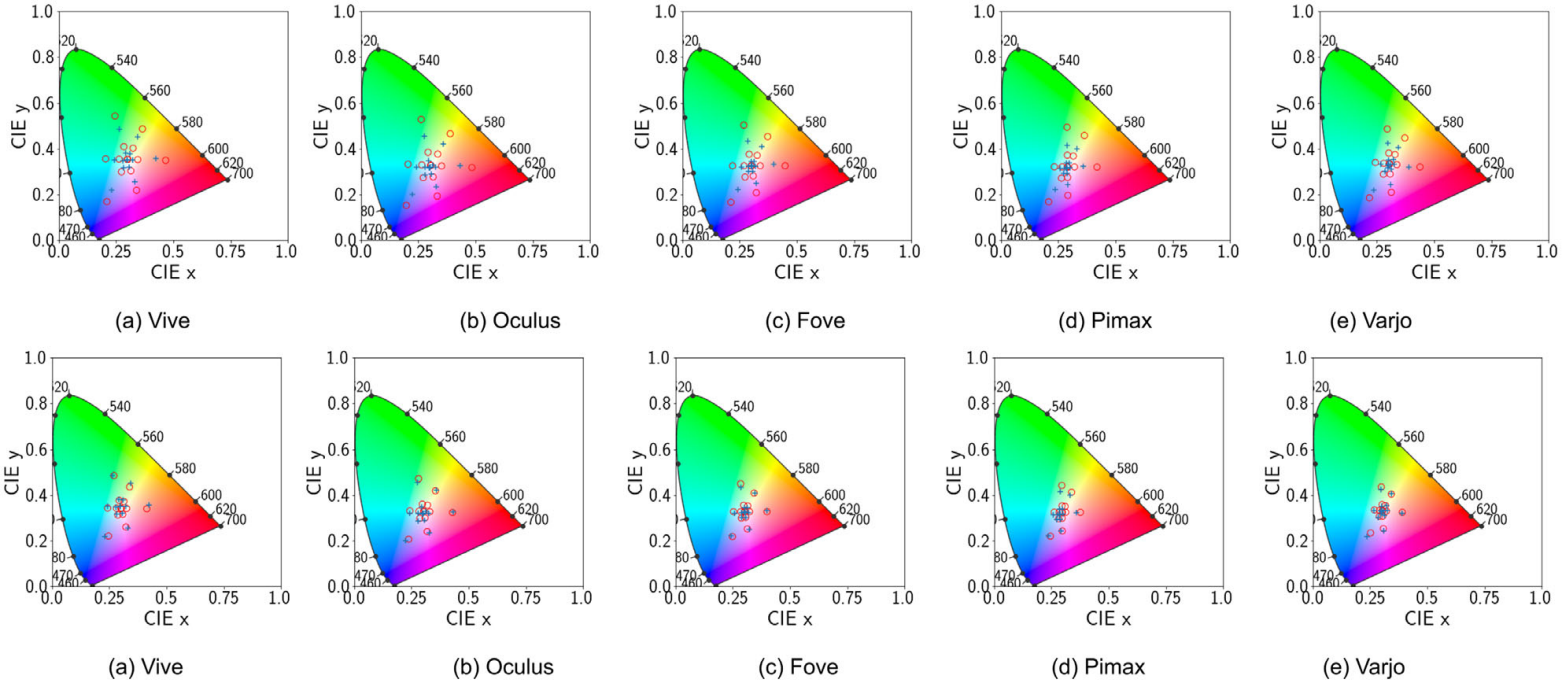
Disabled tonemapping. CIE 1931 Chromaticity diagram with ‘x’ denoting actual device output for different points on the cubes discussed earlier.‘o’ denotes the theoretical position of the actual RGB values using the calibration matrix. The top row shows uncorrected thoretical positions of the cube RGBs, while the bottom row shows gamma corrected positions.

Figure 13 shows how the plotted *Lxy*_*measured*_ and *Lxy*_*predicted*_ do not align perfectly, confirming that standard calibration is affected by the absence of gamma correction. We, therefore, designed the following scheme to apply gamma correction in the XYZ space using α(*RGB*) that converts emissive values to produce the same effect as disabling tonemapping. We apply a function $\tilde{\alpha }\left(xyY\right)$ on *xyY* after converting the predicted *XYZ* = *M*_*corrected*_ · *RGB*^*T*^ to *xyY* using the following equation:

(12) $$\begin{array}{c} R=X+Y+Z\; \end{array}$$

(13) $$\begin{array}{c} x=X/R\; \end{array}$$

(14) $$\begin{array}{c} y=Y/R\; \end{array}$$

(15) $$\begin{array}{c} Y=Y.\; \end{array}$$

For an input *xyY*_*scene*_ and white point *xyY*_*w*_, we use the function $\tilde{\alpha }\left(xyY\right)$ defined as:

(16) $$\begin{array}{c} \tilde{\alpha }\left(xy_{scene}\right)=xy_{w}+\frac{xy_{scene}-xy_{w}}{\left(1+B^{|xy_{scene}-xy_{w}|}\right)}\; \end{array}$$

(17) $$\begin{array}{c} \tilde{\alpha }\left(Y_{scene}\right)=\left\{\begin{array}{cc} x\cdot m_{X\varepsilon [R,G,B]}, & x\cdot m_{X}<t\\ t, & otherwise \end{array}\right..\; \end{array}$$

This correction is formulated by examining the relative positions of the predicted and measured chromaticities. The predicted positions are radially spread more outwards compared with the measured values. Our $\tilde{\alpha }\left(xyY\right)$ function revises the predicted values further inward radially. Figure 20 shows how the function now maps the predicted values very close to the measured ones for *B* = 0.01.

**Figure 14.**
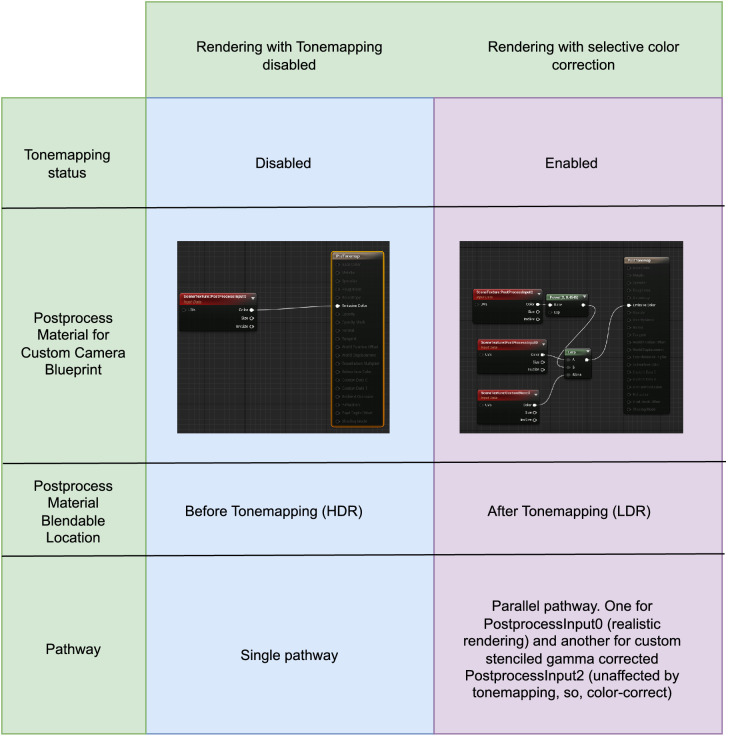
Difference between the two rendering pipelines: i) tonemapping disabled and ii) tonemapping and LDR enabled, gamma corrected.

**Figure 15.**
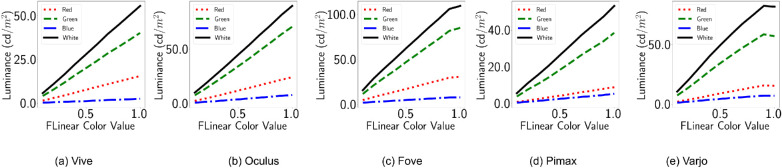
Tonemapping countered. The X axis shows emissive values x in : *RGB*(*x*, 0, 0) for red, *RGB*(0, *x*, 0) for green, *RGB*(0, 0, *x*) for blue, *RGB*(*x*, *x*, *x*) for white. The Y axis shows luminance in *cd*/*m*^2^.

**Figure 16.**
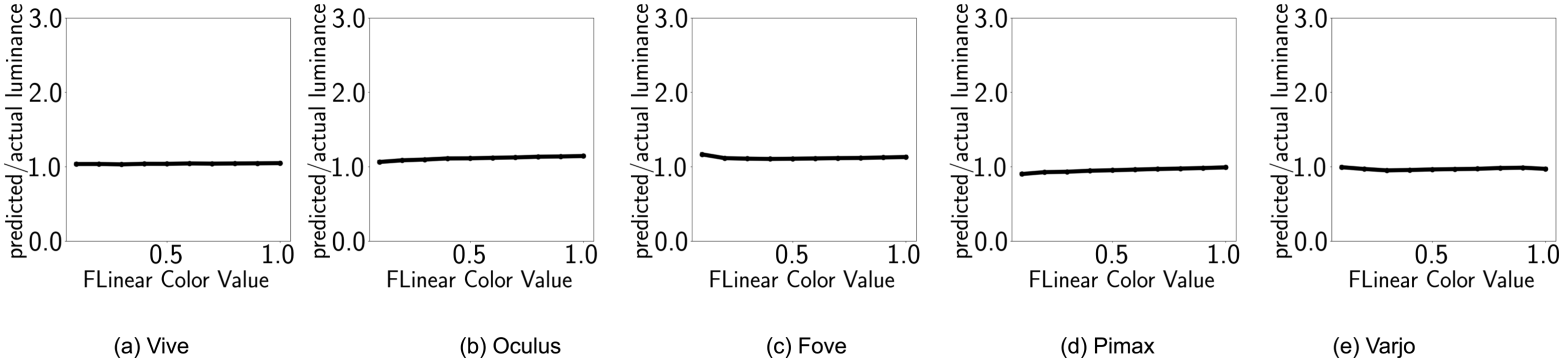
Tonemapping countered. The X axis shows emissive values x while the Y axis shows the ratio (*L*_*R*_ + *L*_*G*_ + *L*_*B*_)/*L*_*W*_.

**Figure 17.**
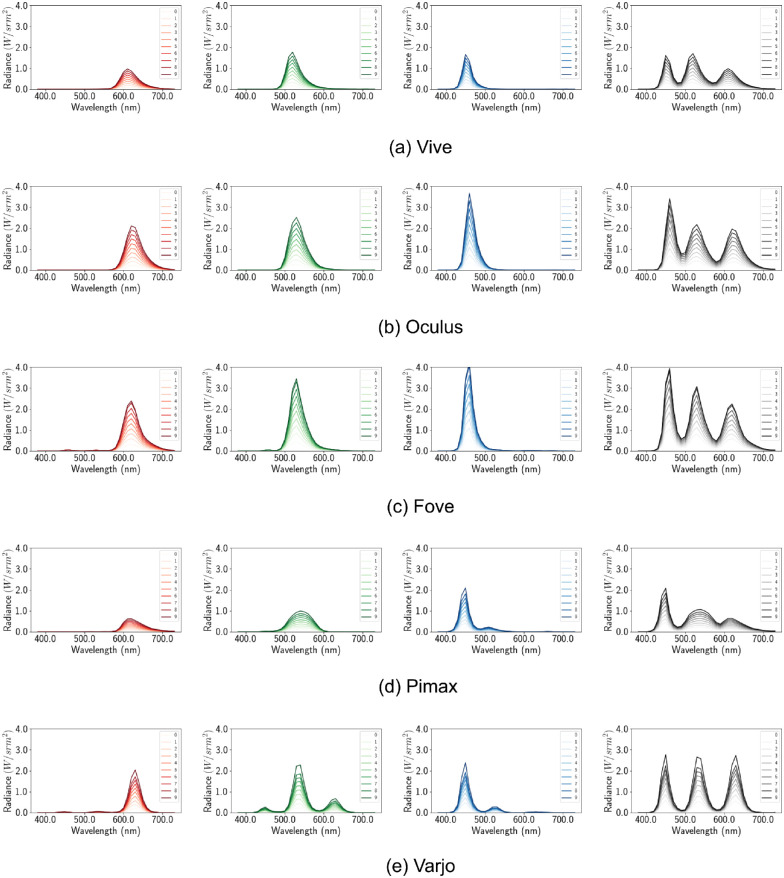
Tonemapping countered. Per channel spectral distribution graph. The X axis denotes the wavelength in nanometers and the Y axis denotes the spectral output at that wavelength. The spectral distribution of different shades of red, green, blue, and white (from left to right) are shown for each device (top to bottom).

**Figure 18.**
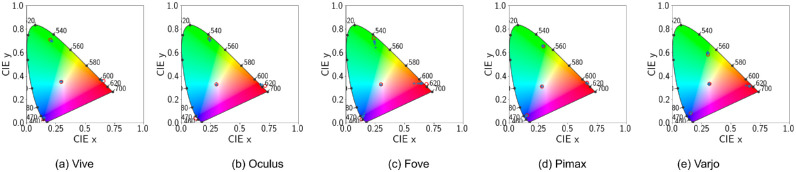
Tonemapping countered. CIE 1931 Chromaticity diagram with ‘x’ denoting actual device output for different shades (0.1 through 0.9) of the primaries. ‘o’ denotes the theoretical position of the actual primaries using the calibration matrix.

**Figure 19.**
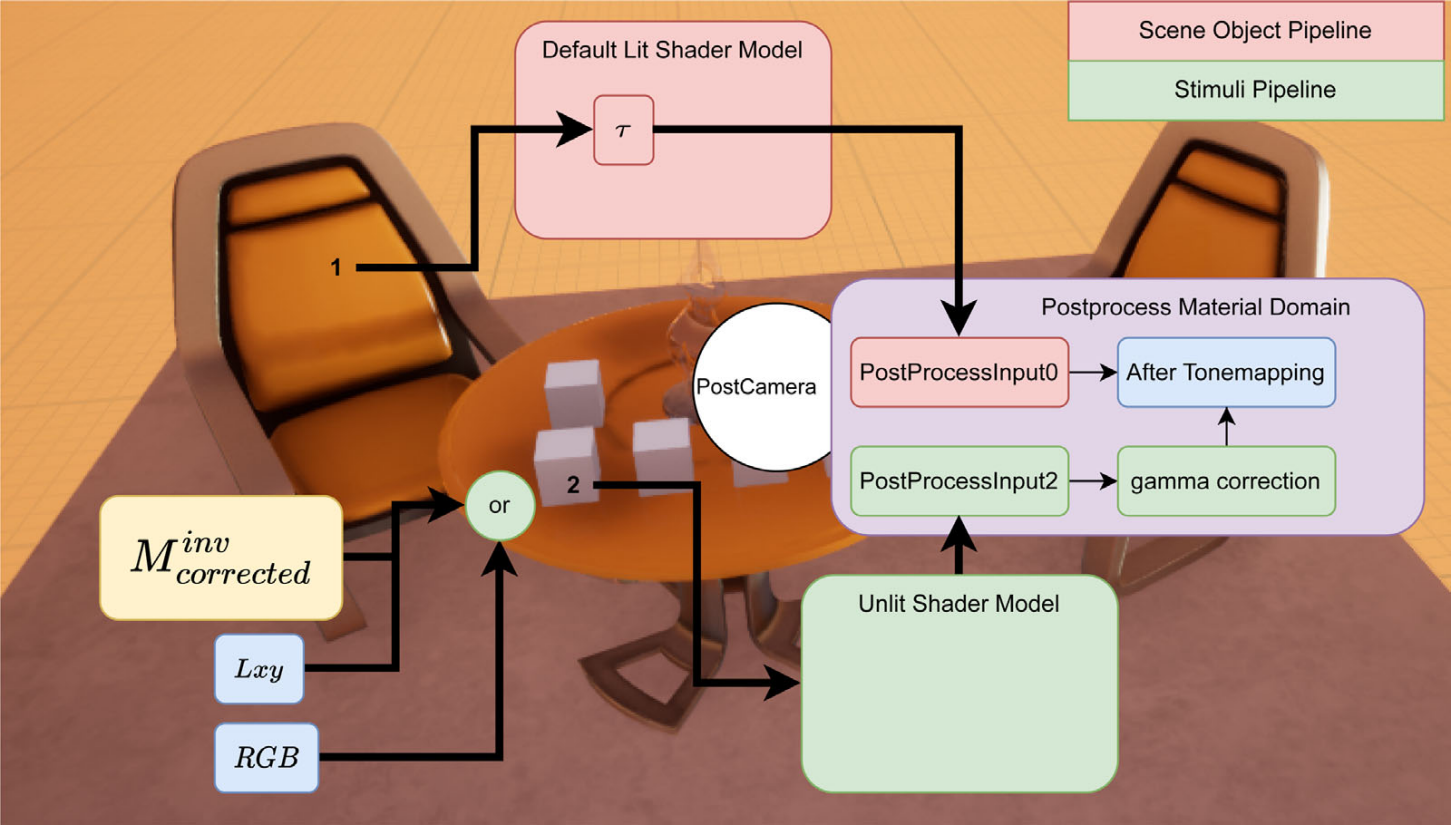
Demonstration of parallel rendering pipelines. (i) Default-lit and (ii) gamma-corrected and unlit.

**Figure 20.**
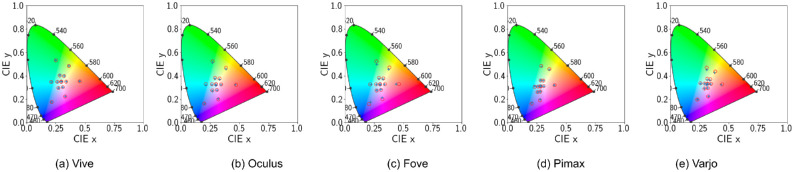
Tonemapping countered. CIE 1931 Chromaticity diagram with ‘x’ denoting actual device output for different points on the cubes discussed earlier.‘o’ denotes the theoretical position of the actual RGB values using the calibration matrix.

The correction shown in Figure 20 is necessary because of the gamma correction being withheld by completely disabling tonemapping and postprocess settings. Essentially, our function is a gamma correction in the chromaticity space.

## Rendering with selective color correction

We repeated the measures and analyses presented in the previous section after re-enabling the tonemapping. However, we still do it with our PostCamera substitution of standard camera. However, we disable all the overrides we made in the previous section, basically reinstating tonemapping. To correct all the effects of tonemapping, we rely solely on the postprocess material now. Instead of using the blendable mode that before tonemapping, we now use after tonemapping so that the material output is now the final render phase. To selectively use custom processing on only the stimuli and leave the rest of the scene with Unreal Engine’s default realistic rendering, we make use of custom render depth pass. This property allows the postprocess material to selectively only apply counter-tonemapping to the stimuli target. In the postprocess material, we use scene texture: PostProcessInput2, which is the scene texture before the tonemapping pass but without gamma correction. We simply correct gamma and it has the intended corrective effect, as will be demonstrated in the following subsections. The differences with previous method is summarized in Figure 14.

### Relationship between input intensity and luminance

Figure 15 shows the luminance (cd/m^2^) corresponding with the input R, G and B channels. The figures demonstrate how our postprocessing material alone alters different HMDs rendering, without changing other postprocess routines. All the displays now exhibit properties of a clipped linear function as the input emissive values. However, one distinction from the clipped linear function of Figure 3 is that now the saturation does not occur immediately. This is due to the proxy shutdown of auto exposure through post process materials. Now, the relationship between input intensity and luminance is similar to when the works of Toscani et al. (2019) disabled postprocessing.

Vive, Oculus, and Pimax show complete linearity in the given emissive ranges in Figure 15. This means that vision experimenters can smoothly change the emissive values to increase the display brightness. Fove and Varjo show slight clipping, showing that the experiments would need to be mindful of the brightness beyond the threshold and either restrict the protocols within the cutoff brightness or account for the two different gradients for luminance variation. Interestingly, Varjo primaries are again a little dimmer compared to the 0.9 shade. Although Fove primaries are not dimmer, the rate of change is diminished.

### Luminance additivity

When postprocess tonemapping is enabled and our α(*RGB*) function is applied to counteract tonemapping for stimuli, luminance additivity is reinstated (Figure 4). For different devices, the ratio of predicted (sum of individual channels) and measured luminance are shown in Table 7. Table 7 and Figure 16 demonstrate that all the displays are now showing almost perfect additivity, whereas OLED displays are very slightly off. Table 8 shows the corresponding values of *m*_*X*_, *t* for each HMD.

**Table 7.** Tonemapping countered: Luminance ratios.

	HTC Vive	Oculus	Fove	Pimax	Varjo
(*L*_*R*_ + *L*_*G*_ + *L*_*B*_)/*L*_*W*_	1.04	1.11	1.12	0.95	0.97

**Table 8.** Tonemapping countered: Slope and threshold for modeled relationship between luminance and emissive values.

	HTC Vive		Oculus		Fove		Pimax		Varjo	
	*m*_*X*_	*t*	*m*_*X*_	*t*	*m*_*X*_	*t*	*m*_*X*_	*t*	*m*_*X*_	*t*
Red channel	15.5	15.5	24	24	30	30	8.7	8.8	17.3	15.4
Green channel	40	40.2	67.2	70.5	87.8	85	37	38.5	67	57
Blue channel	2.5	2.5	7	7	7.3	7.8	5	5	8	7

Whereas Fove used to be the dimmest display in the previous settings, now Fove is one of the brightest displays. It is now seven times as bright as before. Brightness of all HMDs have increased in the current settings, while the dimmest point has remained similar. This results in the overall increase in dynamic range of the system. Transforming Fove from one of the worst devices to render HDR images to one of the best HMDs for that purpose. However, the difference would be more indicative of true potential with auto exposure disabled in the normal camera settings.

### Channel constancy

By applying our corrective material, the new distributions have a higher peak corresponding with the overall increase in luminance (Figure 17). Moreover, constant scaling factors *c* across channels of a device represent multiplicative spectral profile and channel constancy. Table 9 shows the least squared error is consistent with our expectations of channel constancy.

**Table 9.** Tonemapping countered: Channel constancy scaling.

	HTC Vive	Oculus	Fove	Pimax	Varjo
*c*_*red*_	0.012	0.028	0.052	0.021	0.028
*c*_*green*_	0.013	0.026	0.046	0.021	0.012
*c*_*blue*_	0.009	0.019	0.041	0.024	0.026

In Figure 18, we see that the drift in Fove HMD for the chromaticity coordinates of intermediate shades of the whites and primaries is still present. All the primaries theoretical positions now reside within the chromaticity diagram and except for Fove, agree perfectly with measurements.

### Calibration test

With the postprocessing routines re-enabled and application of corrective material, the HMDs now conform to standard calibration procedure. Again, we tested its accuracy by rendering the cube corners. We used the newly calibrated *M*_*corrected*_ to compute the nominal values $XYZ=M\cdot RGB_{cubes}^{T}$ and converting to *Lxy* space. In Figure 20, we see how the measured values and nominal values are very close, indicating that it is possible to control the color of the emitted light with our strategy. Figure 8 shows the rendering pipeline and how the input emissive or chromaticity values are interpreted to render accurate pixels to the HMD

## Discussion

**Figure 21.**
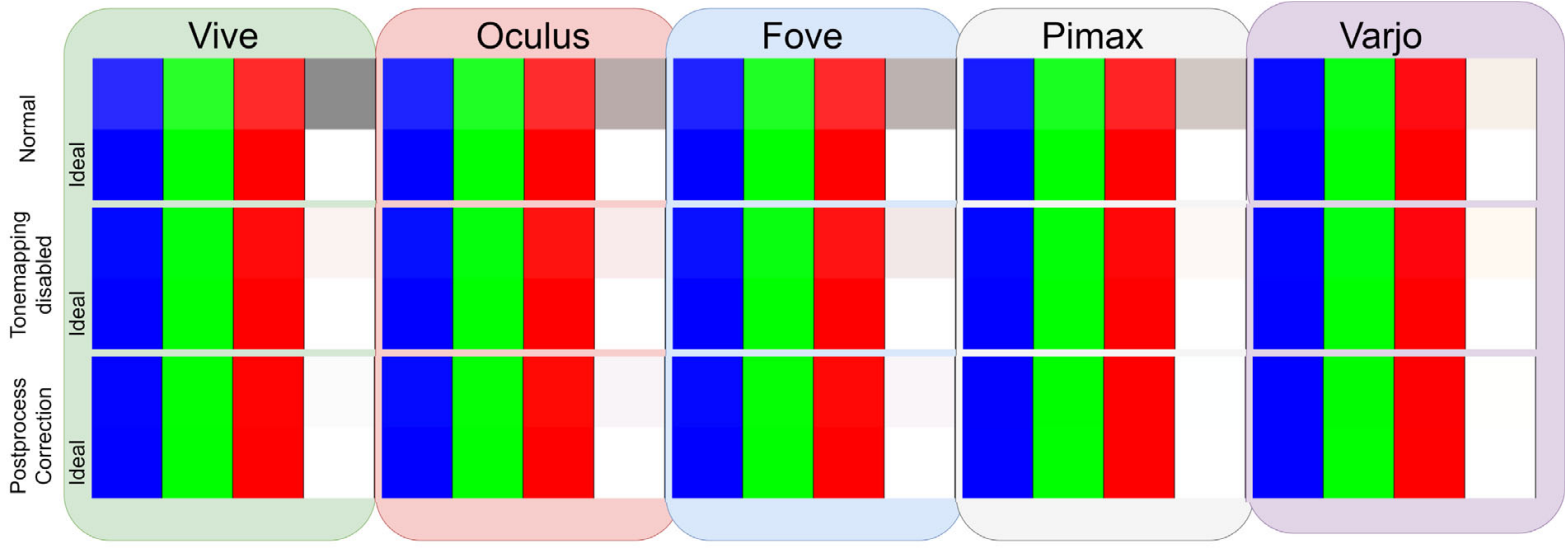
Approximate visualization of the primaries.

**Figure 22.**
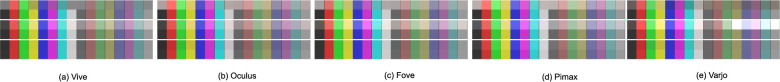
(From left to right: Vive, Oculus, Fove, Pimax and Varjo.) Cube sRGB representation where the top rows are normal settings, middle rows are tonemapping disabled setting and the bottom rows are postprocess corrected settings.

We have established that it is not necessary to disable level wide tonemapping and postprocess settings for color accuracy. Using our hybrid approach permits vision scientists to show color accurate stimuli in real-world settings. This ensures that components of default behavior of Unreal Engine such as auto exposure, high dynamic range, tonemapping etc can be retained for real-world objects while assigning objects of interest with separate graphics render path for color accuracy. This is further demonstrated in the Figure 21 where the primaries of all the HMDs align to the ideal red, green and blue. In the normal settings, the primaries appear more washed out and the whites appear darker for all HMDs. In the settings with tonemapping disabled, the colors appear closer to ideal but slightly off due to absence of gamma correction. The results are even more apparent when we look at the visualization of the cube RGBs in Figure 22 described earlier. The normal settings clearly appear washed out. Although Varjo still shows washed out effect in the tonemap disabled settings, the rest of the HMDs only have a slightly elevated brightness owing to uncorrected gamma.

**Figure 23.**
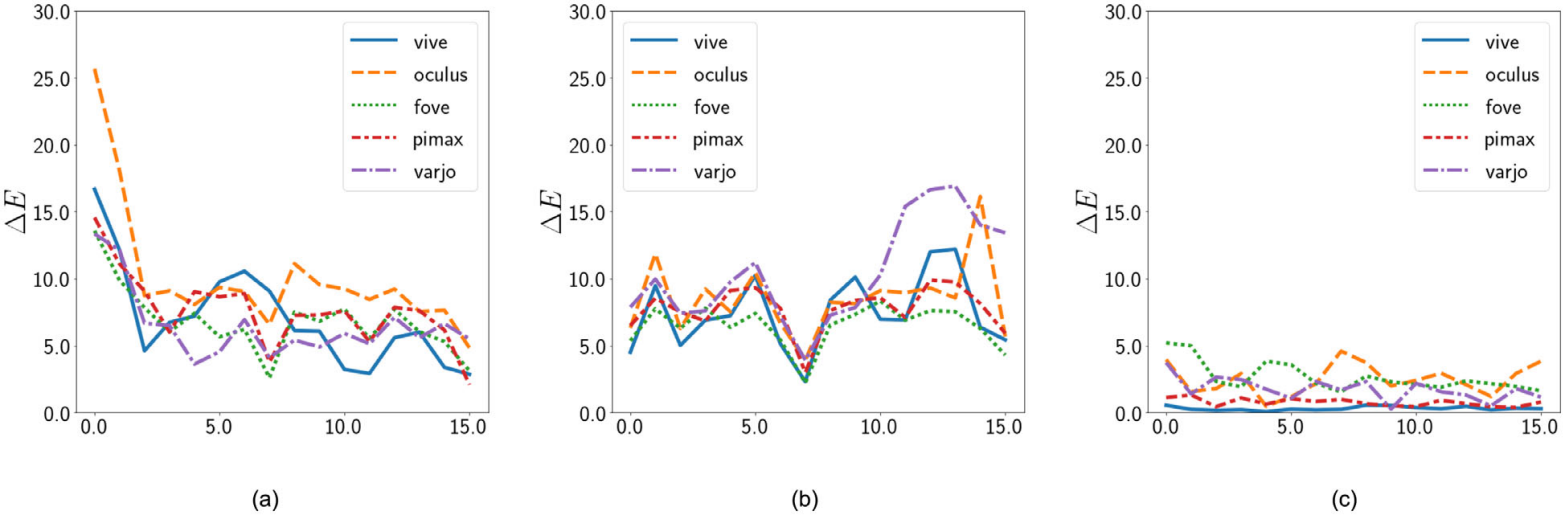
Perceived error. (From left to right Standard postprocessing, disabled tonemapping, and our selective correction approach). The X axis shows index of the cube colors and the Y axis shows the corresponding Δ*E* perceived error.

Table 10 shows how different the peaks of the spectral distributions are for each of the HMDs, which contribute to the overall change in the appearance and chromaticity coordinates of the primaries. Finally, in Figure 23, we can see the Δ*E* (Brainard, 2003) perceived color difference for each of the discussed approaches for the cube colors. Our approach clearly shows significantly lower Δ*E*. Values less than 1.0 is imperceptible to human eyes, and Vive shows the least perceptible difference in our approach, closely followed by Pimax. Values between 1 and 2 indicate differences perceptible through close observation between 2 and 10 are perceptible at a glance. This indicates that for most of the colors, close observation is required to perceive differences when VR devices are calibrated using our approach. Indices 7 and 15 show white and gray points, which are brought closer together when postprocessing is disabled, compared with standard. Overall, the line graph shows that standard postprocessing has an easily perceptible color difference compared with input and the variability between devices is high. However, when the postprocessing routines are disabled, the interdevice variability reduces while the perceived errors converge.

**Table 10.** Wavelength in nanometer of the peak spectral distribution for each primaries.

	HTC Vive	Oculus	Fove	Pimax	Varjo
λ_*red*_	610	620	620	610–620	630
λ_*green*_	520	520	530	540	540
λ_*blue*_	450	460	460	450	450

## Conclusion

Future studies that leverage different rendering pathways laid out in this current work should reveal how this impacts results of color vision research with VR-HMDs. Furthermore, this should help researchers pick the ideal HMD for their particular application. Their are still limitations to the brightness and color gamut achievable by any particular HMD, but with careful consideration of display properties and graphics rendering pipeline, most relevant stimuli can be generated with some of the cheaper HMDs. However, using the two different calibration processes laid out in this work, a wide range of virtual studies can be conducted without requiring the express knowledge of underlying display properties.

## Supplementary Material

Supplement 1
