# Supplementary material for: Calibration of head mounted displays for vision research with virtual reality
Source: J Vis. 2023 Jun 14;23(6):7. doi: 10.1167/jov.23.6.7 (PMC10278547; doi:10.1167/jov.23.6.7)
Supplement: Supplement 1 [file jovi-23-6-7_s001.pdf]

Color Calibration Documentation

Prerequisite:
Color calibration fo a VR display requires the following sets of hardware:

1. Spectroradiometer
2. VR Headset stand

The minimum requirements for the computing hardware should be determined by the recommendation of the specific VR headset manufacturer.

The following software packages need to be installed:

1. Unreal Engine v4.27 -> link
2. Visual Studio 2022 -> link
3. Steam and SteamVR -> link, link
4. Matlab and measureDisplay packages -> link, link
5. Download https://github.com/Znasif/ColorCalibration

After installation, please follow the next steps to calibrate and use your VR headset for accurate color reproduction.

Steps for Calibration:

1. Right click on the ColorCalibration.uproject file and select “Generate visual studio project files”. This will generate the necessary files for project execution. Open the “ColorCalibration.sln” in visual studio and run the project.

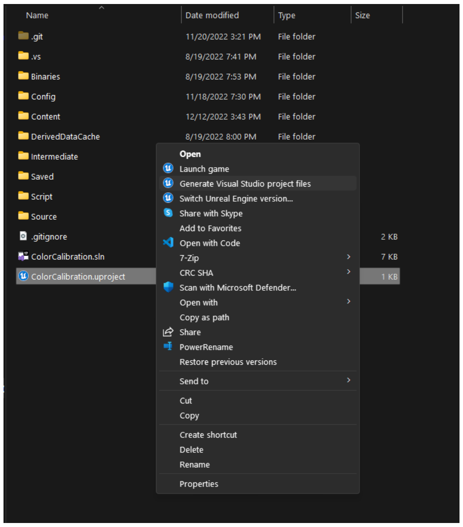

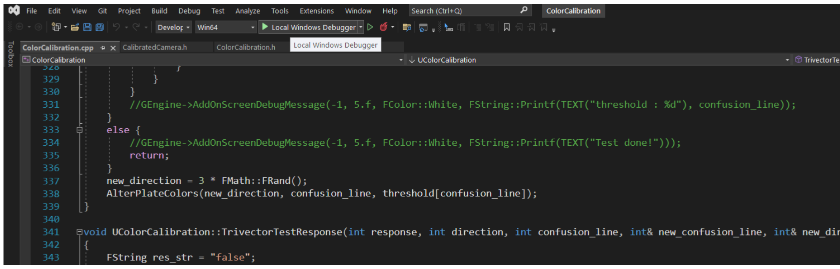

2. When the STEAMVR application opens, click on the menu button with three lines. From the dropdown menu, select “Display VR View”. Display VR View. This will help you to see what the participant is viewing at that particular time.

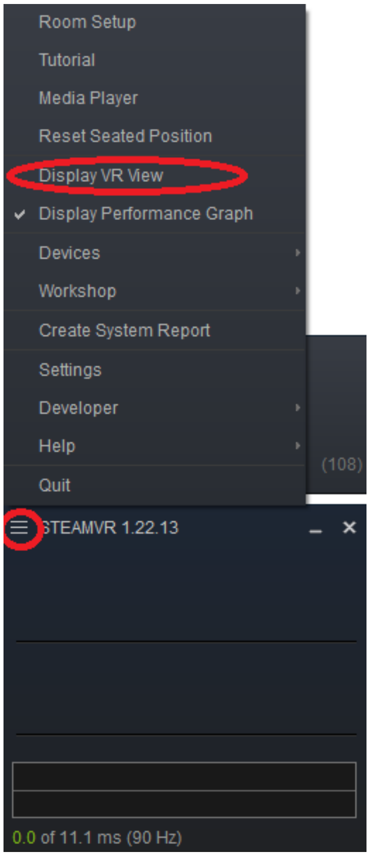

3. In the Unreal Engine Editor content menu find the level called JustStimuli. We will be using this level to obtain the calibration data about the selected VR display.

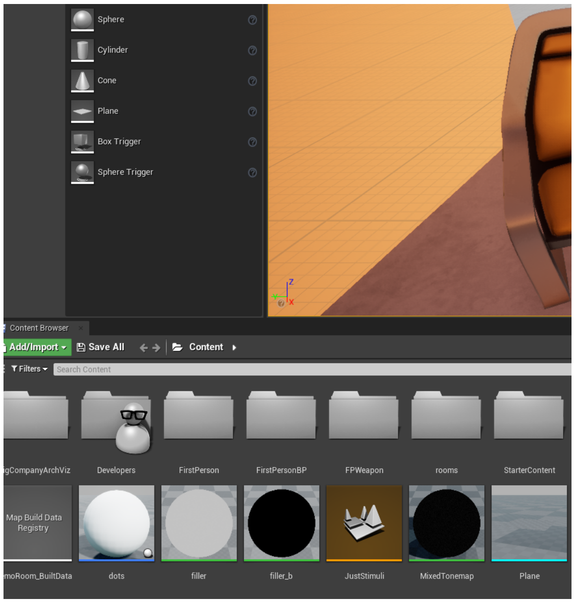

4. Connect the spectroradiometer to the computer and launch the measureDisplay.m file in MATLAB.

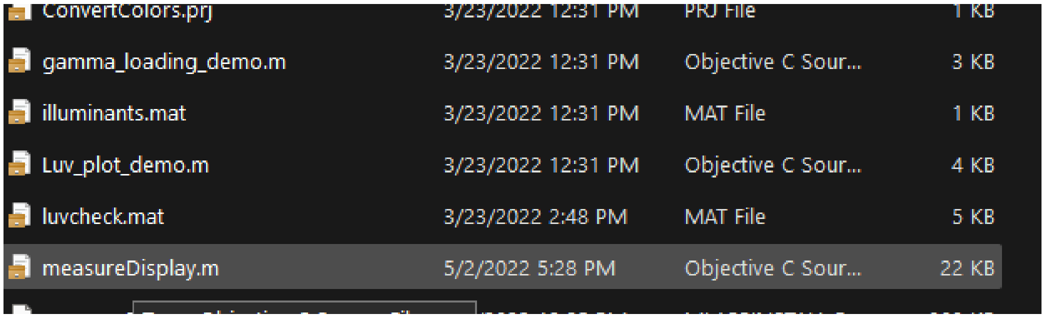

5. Now open the level blueprint of the JustStimuli level.

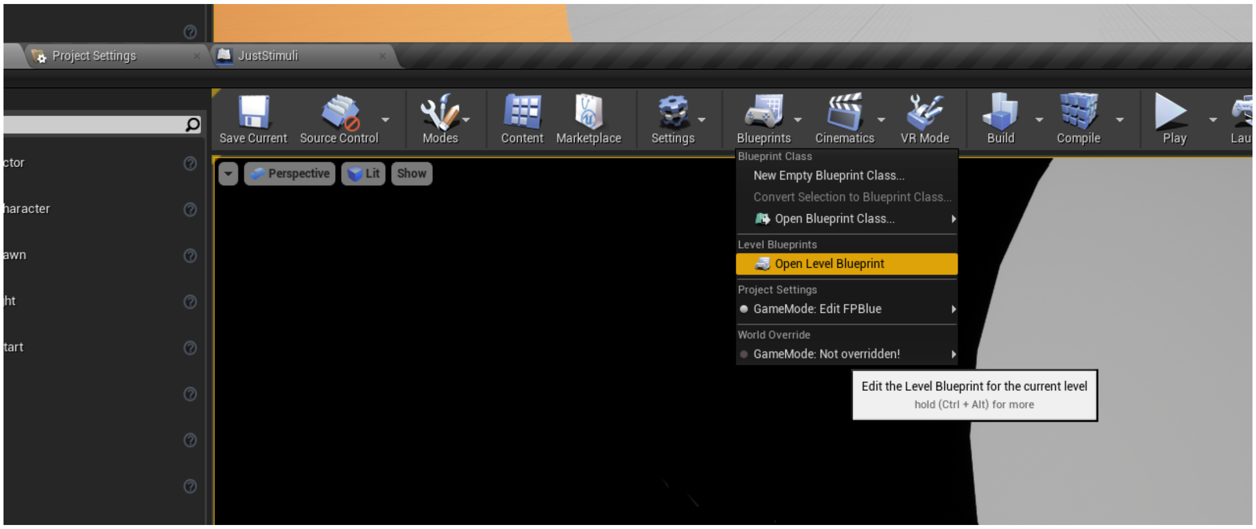

6. You will see the following options: collecting primaries, stepped brightness, cubed big and small (details in the paper). Connect the up route to whichever data you wish to collect. For calibration we need primaries and stepped brightness.

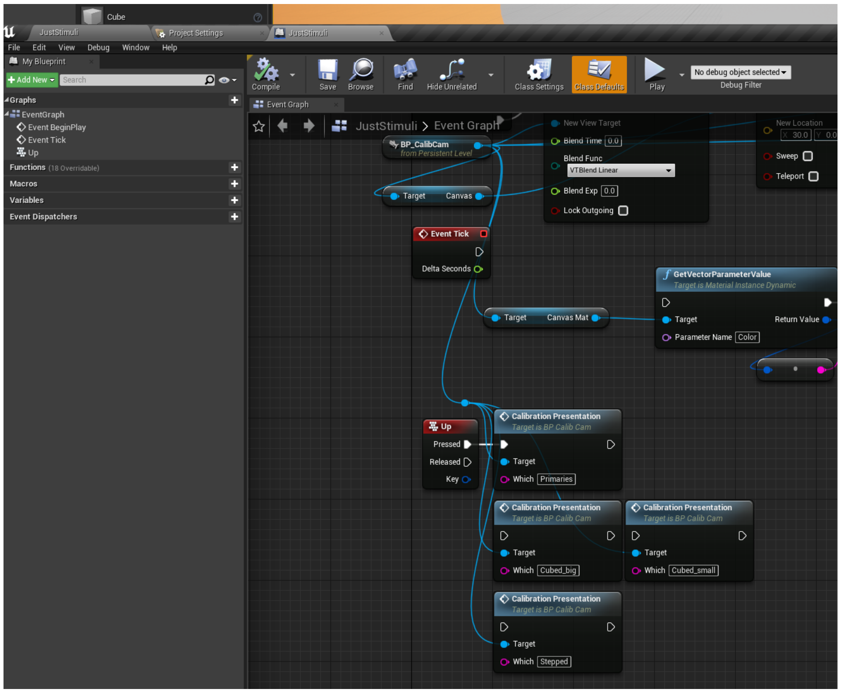

7. Calibrate the spectroradiometer.
8. Next, select the VR preview and hold the spectroradiometer against the screen of the VR display as flush as possible and normal to the screen at the center. A VR stand is very useful to place the two hardware in a reliable manner. Run the matlab script and it will record the screen chromaticity and luminance. Gather the data into a csv file. The primaries file is read through the readPrimariesFromCSV function in Unreal and will create the calibration matrix for subsequent manipulations.

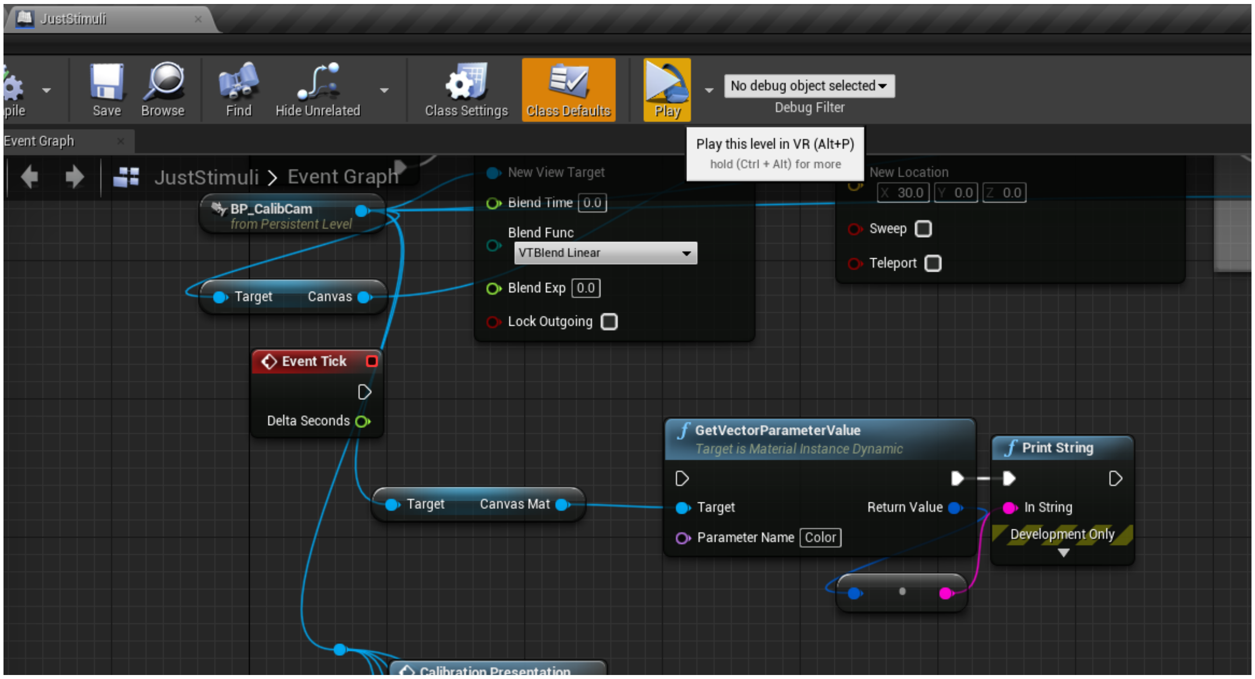

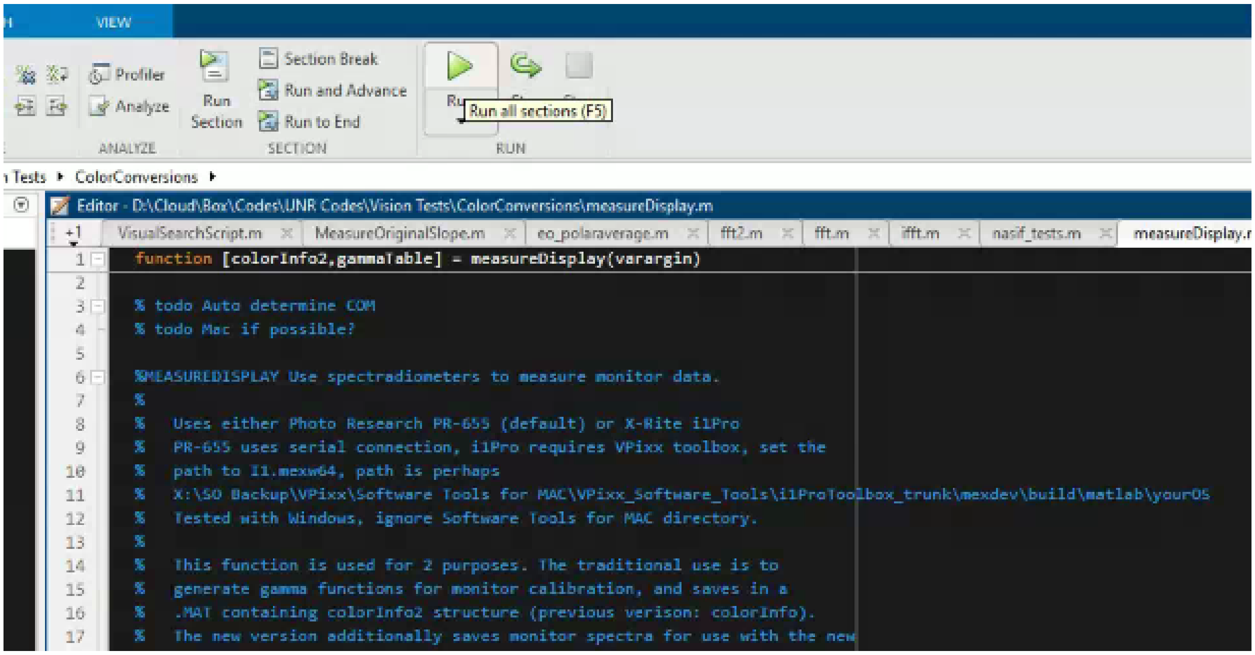

Steps for Using BP_CalibCam:

1. BP_CalibCam has all the necessary components to view color correct output in the VR display. To be able to view color correct elements, all the user needs to do is add a BP_CalibCam object to the level, set view target to it and apply unlit shader model to stimuli material.

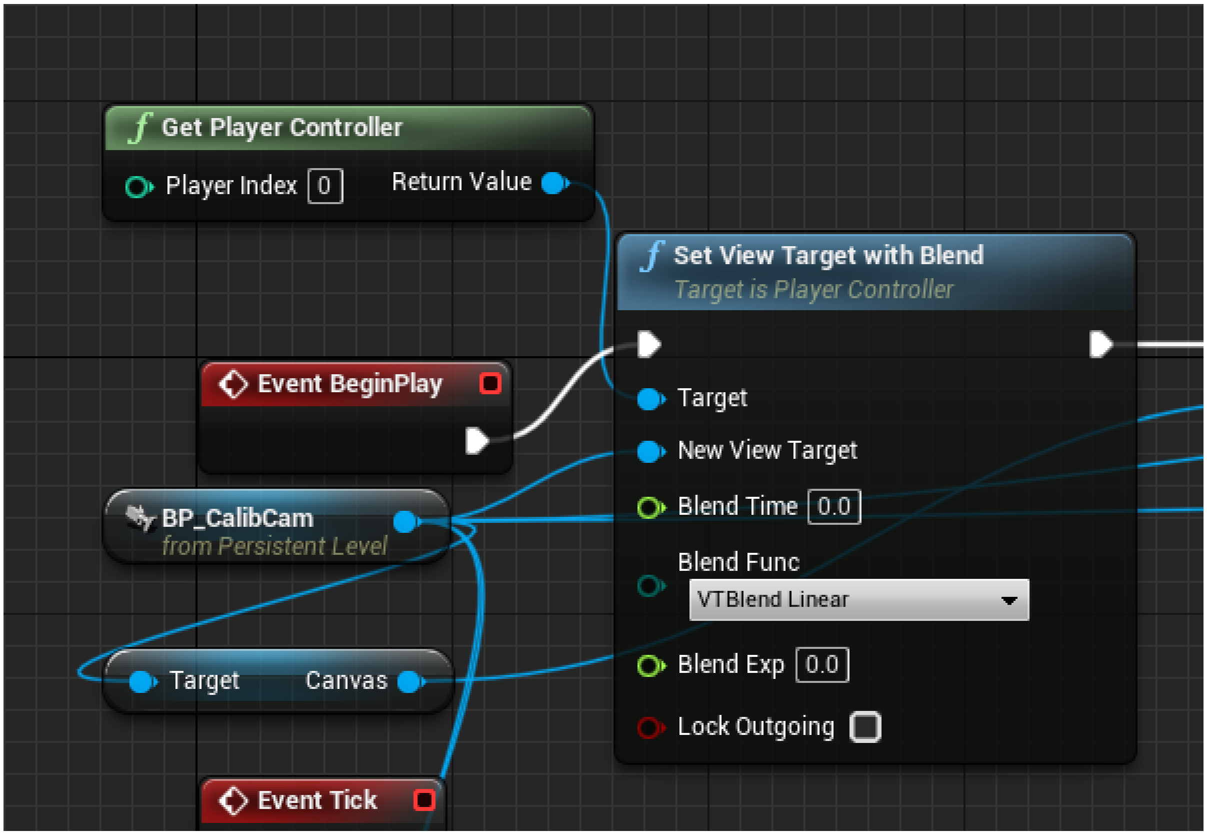

2. To turn the stimuli material to color correct follow the following steps:
    a. Use FColor_Luv or FColor_lxy to describe the chromaticity and luminance of the stimuli.

b. Use convertFromLuvtoRGB which uses the described matrix to convert to RGB and apply the output to unlit shader material and emissive output

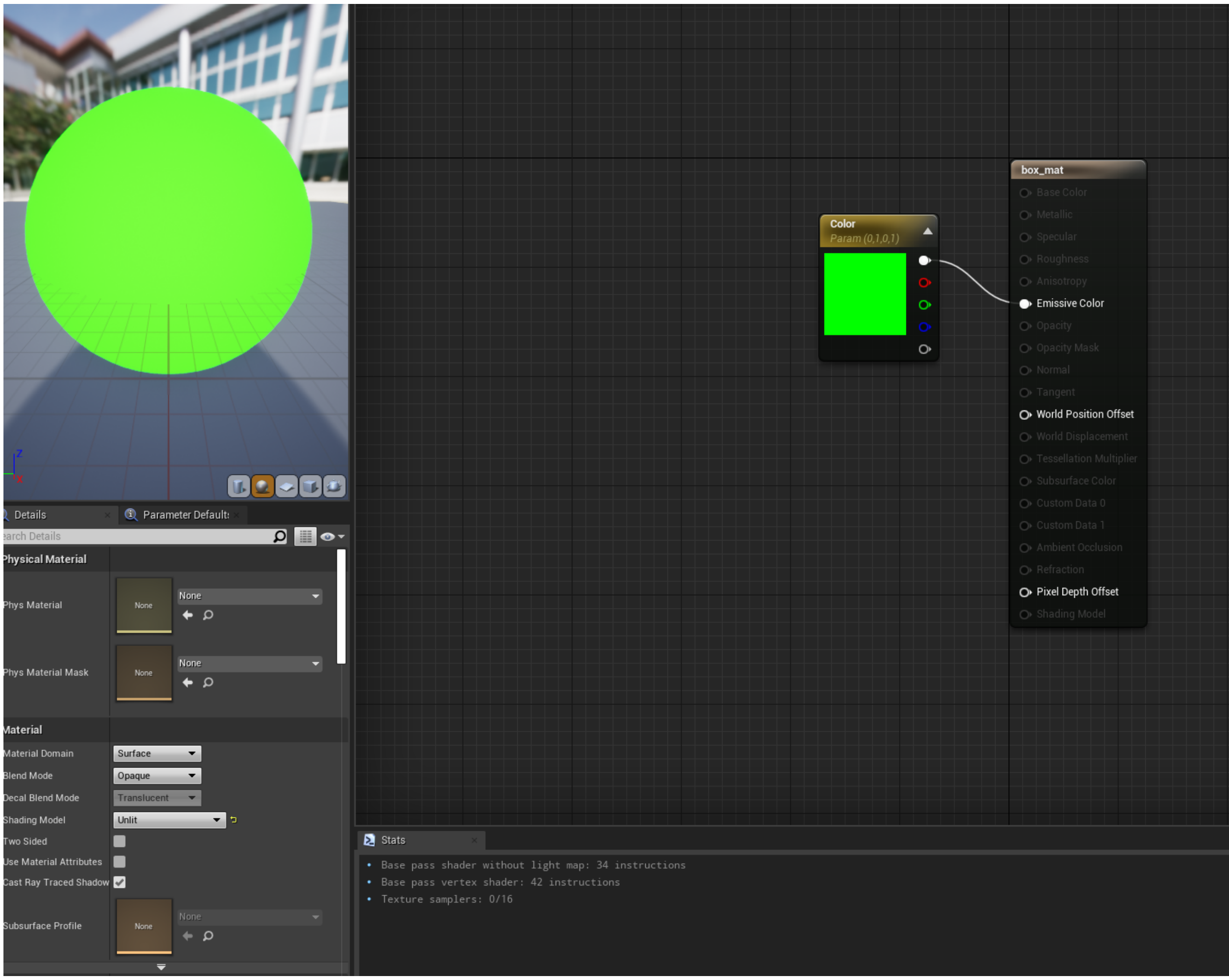

c. If custom stencil is used to tag the object, it can either perform photorealistically with unaltered pipeline or perform color correctly.

d.

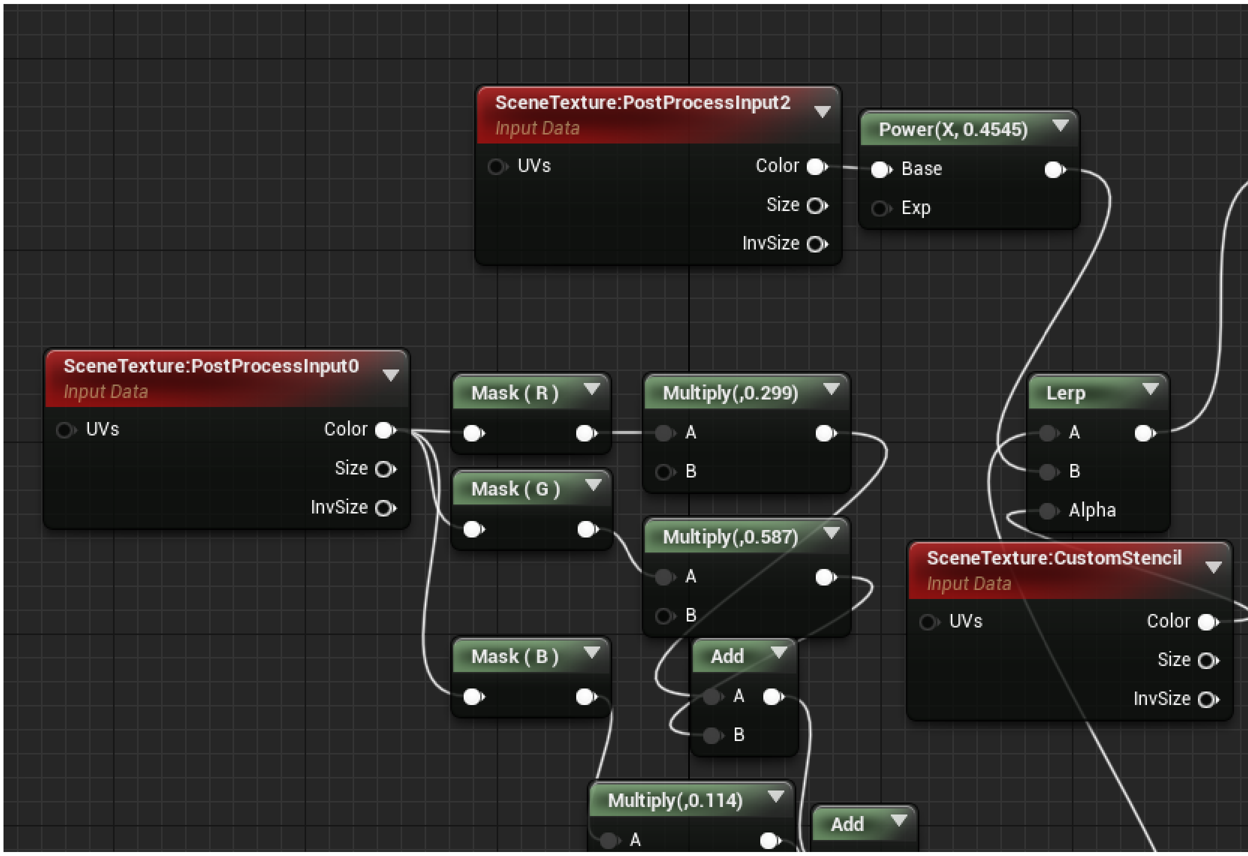
SceneTexture:PostProcessInput2
Input Data
UVs
Color
Size
InvSize
Power(X, 0.4545)
Base
Exp
SceneTexture:PostProcessInput0
Input Data
UVs
Color
Size
InvSize
Mask (R)
Mask (G)
Mask (B)
Multiply(,0.299)
A
B
Multiply(,0.587)
A
B
Add
A
B
Multiply(,0.114)
A
Add
Lerp
A
B
Alpha
SceneTexture:CustomStencil
Input Data
UVs
Color
Size
InvSize
